# Cations Regulate Membrane Attachment and Functionality of DNA Nanostructures

**DOI:** 10.1021/jacs.1c00166

**Published:** 2021-05-07

**Authors:** Diana Morzy, Roger Rubio-Sánchez, Himanshu Joshi, Aleksei Aksimentiev, Lorenzo Di Michele, Ulrich F. Keyser

**Affiliations:** † Cavendish Laboratory, University of Cambridge, JJ Thomson Avenue, Cambridge CB3 0HE, United Kingdom; ‡ Department of Physics, University of Illinois at Urbana−Champaign, 1110 West Green Street, Urbana, Illinois 61801, United States; § Beckman Institute for Advanced Science and Technology, University of Illinois at Urbana−Champaign, 405 North Mathews Avenue, Urbana, Illinois 61801, United States; ∥ Department of Chemistry, Molecular Sciences Research Hub, Imperial College London, London W12 0BZ, United Kingdom

## Abstract

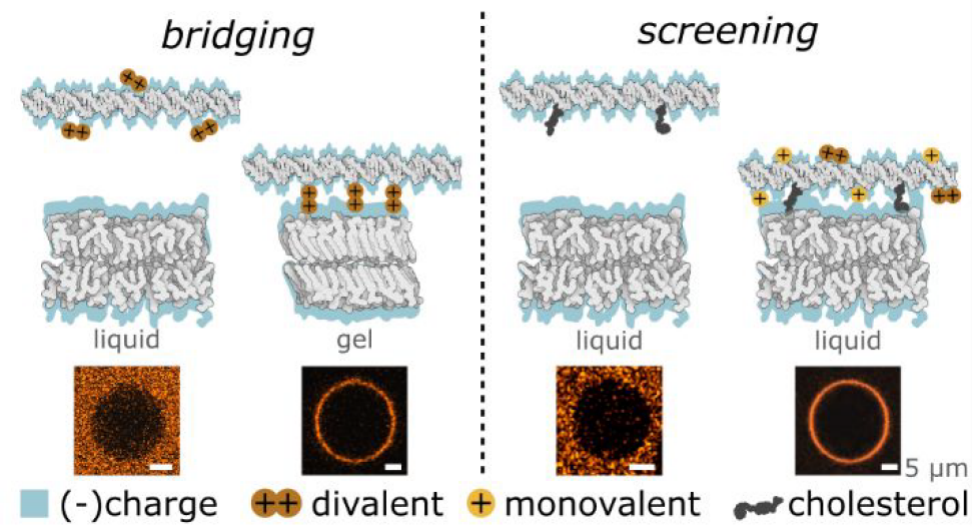

The interplay between nucleic acids and lipids underpins several key processes in molecular biology, synthetic biotechnology, vaccine technology, and nanomedicine. These interactions are often electrostatic in nature, and much of their rich phenomenology remains unexplored in view of the chemical diversity of lipids, the heterogeneity of their phases, and the broad range of relevant solvent conditions. Here we unravel the electrostatic interactions between zwitterionic lipid membranes and DNA nanostructures in the presence of physiologically relevant cations, with the purpose of identifying new routes to program DNA–lipid complexation and membrane-active nanodevices. We demonstrate that this interplay is influenced by both the phase of the lipid membranes and the valency of the ions and observe divalent cation bridging between nucleic acids and gel-phase bilayers. Furthermore, even in the presence of hydrophobic modifications on the DNA, we find that cations are still required to enable DNA adhesion to liquid-phase membranes. We show that the latter mechanism can be exploited to control the degree of attachment of cholesterol-modified DNA nanostructures by modifying their overall hydrophobicity and charge. Besides their biological relevance, the interaction mechanisms we explored hold great practical potential in the design of biomimetic nanodevices, as we show by constructing an ion-regulated DNA-based synthetic enzyme.

## Introduction

Understanding the interplay between nucleic acids (NA) and lipids is crucial for unravelling various biological processes as well as for the development of techniques and devices in bioengineering and nanomedicine. For instance, DNA–membrane contacts have been functionally implicated in the replication of genetic material in both prokaryote and eukaryote cell cycles,^1^ while synthetic biology harnesses NA–lipid interactions in recombinant DNA technologies for the purpose of cell transformation.^2−4^ Similarly, NA–lipid formulations are critical in (bio)medicine for gene therapy^5,6^ and vaccine technologies. In fact, mRNA-loaded lipid nanoparticles form the basis of the first COVID-19 vaccines licensed for human distribution,^7,8^ illustrating the social benefits that the rational engineering of NA–lipid formulations can bring.

In addition, the understanding of DNA–lipid interactions underpins our ability to engineer and interface lipid membranes with synthetic DNA nanostructures, designed to replicate key functionalities of biological membrane proteins, including the ability to remodel bilayers,^9−11^ induce lipid flipping^12,13^ and regulate ion transport,^14−18^ membrane–membrane adhesion,^19,20^ and fusion.^21−23^

Electrostatic forces are key to NA–lipid interactions, in view of the strong negative charge of nucleic acid backbones^24^ and the diverse charge architectures found in lipid headgroups.^25^ Adding to this already intricate picture is the fact that these nanosystems exist in complex solvent conditions, where ions of different valency and size are present at varying concentrations, and can screen or enhance Coulomb interactions.^26−29^ Accounting for the effect of (physiological) ions is therefore critical to inform the design of both NA–lipid formulations and membrane-active DNA nanodevices. The latter, in particular, often feature complex and programmable morphologies, charge distributions, and chemical modifications,^9,13,30^ whose coupling with the electrostatic effects mediated by cations could unlock novel functionalities.

Here, we unambiguously identify routes through which ions modulate the electrostatic interactions between DNA and bilayers. We demonstrate that the action of cations can be employed to dynamically program DNA–lipid interactions through its coupling with system parameters such as lipid phase, the architecture of the DNA constructs, and the presence of chemical modifications.

Our experiments focus on the general scenario in which short double-stranded (ds, duplex) DNA interacts with the zwitterionic phosphatidylcholine (PC) lipid bilayer. The stability of dsDNA probes over broad ionic-strength ranges^31,32^ allows us to disentangle the effects of membrane–DNA interactions from ion-dependent structural changes. Zwitterionic lipids are among the most common components of synthetic and biological membranes,^33^ and while they carry no net charge, the different accessibility of the charged moieties on the headgroup can result in Coulomb interactions with other macromolecules.^34−37^

First, we report on the emergence of adhesive interactions between gel-phase membranes and DNA in the presence of divalent cations, while no adhesion is observed toward liquid-phase membranes or in the presence of monovalent cations alone. We ascribe this behavior to phase-dependent cation bridging, as confirmed by means of all-atom molecular dynamics (MD) simulations. We then modulate membrane attachment of DNA nanostructures with parameters influencing bilayer phase, including temperature and sterol content.

We then show that even for liquid-phase bilayers, cations still strongly influence DNA–membrane interactions by screening Coulomb repulsion. To accurately probe this effect, we modify the dsDNA probes with cholesterol moieties inducing attractive DNA–membrane forces that compete against charge repulsion.^12,13,16,17^ Required to screen electrostatic repulsion and enable binding, cations are shown to influence the balance between the competing forces in a way that depends on the charge-to-hydrophobicity ratio of the DNA nanostructures. Besides allowing us to quantify the impact of charge screening, the latter observation identifies yet another mechanism though which the interactions between lipids and DNA can be fine-tuned.

We applied our findings by exploiting ion-dependent membrane attachment to program reversible insertion and activation of a synthetic DNA enzyme. This proof-of-concept experiment exemplifies the direct applicability of our observations to the rational design of nanobiotechnological processes, with clear implications for next-generation diagnostic, therapeutic, and synthetic biological tools.

## Results and Discussion

### Cation-Mediated *Bridging* between DNA and Zwitterionic Membranes Is Dependent on Lipid Phase

**Figure 1:**
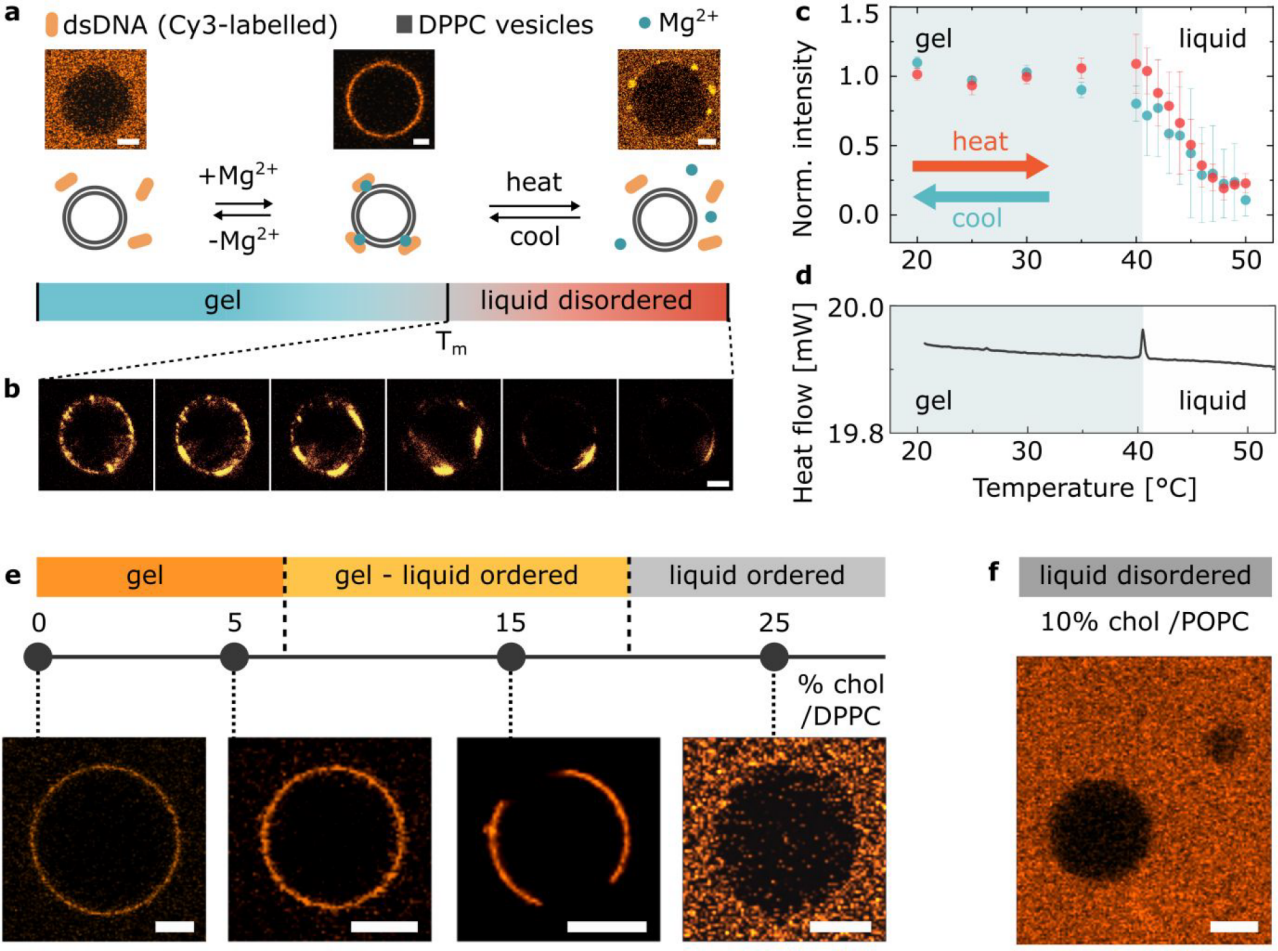
Cations mediate bridging between DNA and gel-phase PC bilayers. (a) Representative confocal micrographs and schematic depiction of the interaction between DPPC GUVs and Cy3-labeled dsDNA in buffers with and without magnesium salt added, as observed at room temperature and upon heating above the phase transition temperature (*T*_m_) of lipids. Scale bars: 5 μm. (b) Representative confocal micrographs of DPPC GUVs showing gradual detachment of the dsDNA with the temperature increasing above *T*_m_. Scale bar: 5 μm. (c) Temperature dependence of the attachment of DNA constructs to DPPC GUVs recorded via fluorescence upon heating (red points). The turquoise data points were collected on cooling the sample down, illustrating reversibility of the process. The error bars indicate the standard deviation from three independent experiments. (d) Differential scanning calorimetry (DSC) plot of DPPC large unilamellar vesicles (LUVs) incubated with dsDNA in the presence of Mg^2+^. The position of the peak indicates the transition temperature (*T*_m_) of the membrane. An analogous curve was obtained for lipid samples lacking DNA or cations (Figure S4). (e) Dependence of the DNA attachment on bilayer phase, as modulated by changing cholesterol molar fraction in DPPC/cholesterol binary mixtures. For GUVs displaying gel–L_o_ phase coexistence (cholesterol/DPPC molar ratio of 15%) the DNA is localized in parts which presumably correspond to the gel-phase domains (Figure S10). Scale bars: 10 μm. (f) Representative confocal micrograph demonstrating the lack of DNA attachment on liquid disordered POPC/cholesterol GUV. Scale bar: 10 μm.

To quantify DNA–membrane attachment, we employed optical assays and the use of confocal microscopy, as illustrated with representative micrographs in Figure 1a. Giant unilamellar vesicles (GUVs) were chosen as a platform to experimentally probe DNA–membrane interactions. We incubated GUVs prepared from DPPC lipids with short, fluorescently labeled 36 bp DNA duplexes (Figure S1, oligonucleotides sequences in Table S1) using buffers with and without added salt (buffer compositions detailed in section S3 of the Supporting Information).

In the presence of 1 mM Mg^2+^ ions, GUVs at room temperature (≈25 °C) show a bright layer of DNA on their surface. Conversely, in the absence of salts GUVs do not display DNA attachment, even though polyacrylamide gel electrophoresis (PAGE) confirms the stability of the DNA probes in the absence of divalent cations (Figure S2). This hints at an electrostatic origin of the observed behavior and is consistent with previous reports of ion–membrane interactions^34,35^ and DNA–membrane binding mediated by divalent cations.^36−40^

No membrane attachment was observed in the presence of solely monovalent ions (K^+^ and Na^+^), even at 200 mM concentration, while both Mg^2+^ and Ca^2+^ caused DNA to coat the membrane (Figure S3), which suggests that the reported behavior can be attributed to cation bridging, known to be induced only by multivalent ions.^41,42^

The evidence of divalent-ion bridging is consistent with the previously reported decrease in DNA–membrane affinity following the addition of monovalent ions,^43,44^ given that bridging depends strongly on the monovalent-to-divalent ions ratio.^45^ This notion traces a simple route to modulate DNA–membrane attachment, which we have explored in Discussion 1 of the Supporting Information by testing mixtures of monovalent and divalent cations emulating ionic compositions found in key cellular compartments. For instance, we observed that high (100 mM) concentrations of monovalent cations are sufficient to suppress the bridging ability of Mg^2+^ present at 1 mM concentration, but not at 20 mM. The trend can lay foundations for the development of nanostructures responsive to fluctuations in cation concentration due to physiological or pathological changes.^46,47^

Importantly, the melting temperature of DPPC is *T*_m_ = 41 °C^48,49^ (Figure S4), which entails that at room temperature the GUVs display a gel or solid-like phase.^50^ No DNA attachment was observed on GUVs prepared from POPC, which although sharing the same headgroup as DPPC (PC, phosphatidylcholine), form liquid disordered bilayers (L_d_) at room temperature (*T*_m_ = −2 °C) (Figure S5). The difference in DNA attachment between gel and L_d_ PC bilayers hints at the regulatory role of the lipid phase - a concept that has been previously evoked to rationalize the partitioning of DNA structures in phase-separated bilayers.^51,52^

To further elucidate the role of the lipid phase on DNA attachment, a phase transition was induced in samples of DPPC GUVs incubated with the same dsDNA nanostructures and 1 mM Mg^2+^ ions by gradually heating them up from room temperature to well above *T*_m_ (50 °C) during imaging. Below the transition temperature the DNA coating remained uniform. However, as the temperature was increased above *T*_m_ and the liquid phase appeared (Figure S6), we observed the emergence of a patchy DNA distribution, followed by a gradual detachment of the constructs (Figure 1a,b). Figure 1c quantitatively illustrates the temperature dependence of DNA attachment by using the average fluorescence intensity recorded on the GUVs as a proxy. The fluorescence intensity data, and their comparison with the position of the differential scanning calorimetry (DSC) peak in Figure 1d, confirm that DNA detachment initiates at *T*_m_ and proceeds gradually, as the temperature is increased. A control experiment, in which the sample was incubated at constant temperature just above *T*_m_ for an extended period of time, highlights no evolution in the DNA coating density, suggesting that the gradual detachment seen in Figure 1c is not an artifact of a slow desorption kinetics, but rather represents the equilibrium behavior of the system (Figure S7). The ability of the system to reach equilibrium over the time scales relevant to Figure 1c (1 min per data point) is further confirmed by the absence of any significant hysteresis between the curves collected on heating (red) and cooling (turquoise) and by the direct assessment of desorption kinetics, which is found to occur over the seconds time scale (Figure S8). Additionally, we excluded DNA construct destabilization (Figure S9) as a possible effect biasing the observed trend. Data collected on cooling, shown in Figure 1c, illustrate the reversibility of the temperature-dependent DNA attachment.

Besides temperature, the phase of DPPC-based bilayers can be tuned isothermally by altering their composition with the addition of cholesterol. To help disentangling the effect of temperature and bilayer phase on membrane-DNA adhesion, we tested GUVs prepared with cholesterol/DPPC molar ratios of 0%, 5%, 15%, and 25%, which at room temperature display a homogeneous gel phase (0 and 5%), coexistence of gel and liquid ordered (L_o_) phases (15%), and a homogeneous L_o_ phase (25%).^50^ Representative micrographs of these compositions, in the presence of 1 mM Mg^2+^, are shown in Figure 1d, which further confirm that the electrostatics-mediated attachment is only prevalent in the presence of membranes in a gel phase, while no attachment is observed for the L_o_ phase. Notably, the cholesterol itself does not mediate DNA attachment when embedded in a liquid bilayer, given that no DNA adhesion is observed at high cholesterol/DPPC molar fractions or if cholesterol is added to POPC membranes, as shown in Figure 1e.

The data summarized in Figure 1 demonstrate that divalent cations can mediate adsorption of dsDNA to zwitterionic (PC) lipid membranes. However, the effect is only detectable for gel-phase bilayers, while DNA does not adhere to PC bilayers with liquid - either L_o_ or L_d_ - phases. A possible explanation for the observed trends would be a phase-dependent difference in the affinity of divalent cations to the lipid headgroups, which would in turn influence their ability to bridge DNA. To further test this hypothesis, we performed zeta (ξ) potential measurements on both DPPC and POPC large unilamellar vesicles (see Supporting Information Discussion 2). These measurements confirm a clear difference between the surface potentials of DPPC bilayers below and above *T*_m_ in the presence of magnesium, which triggers DNA absorption. However, we identified negative surface potentials in both gel-phase (−12.20 ± 0.22 mV) and liquid-phase (−18.07 ± 1.45 mV) membranes at room temperature. This suggests that although DNA–lipid adhesive interactions may be mediated by divalent cation bridging, factors other than surface charge regulate adsorption of cations onto PC bilayers and thus, DNA adhesion.

We therefore performed all-atom molecular dynamics (MD) simulations to gain direct insights into the mechanism of Mg^2+^-mediated interaction between dsDNA and bilayers. To this end, we constructed several systems, each containing a patch of liquid-phase (DPhPE, DPhPC) or gel-phase (DPPE, DPPC) bilayer, and probed their interactions with Mg^2+^ and dsDNA.

In the absence of DNA, free equilibration simulations of the four membrane systems in high concentrations (100 and 300 mM) of MgCl_2_ solutions showed accumulation of Mg^2+^ ions near the lipid headgroups (Figures S11 and S12). Furthermore, the local concentration of Mg^2+^ ions was found to be considerably higher near the surface of the gel-phase membranes (DPPE and DPPC) than near the surface of the fluid-phase bilayers (DPhPE and DPhPC), indicating a stronger affinity of Mg^2+^ to gel phases.

**Figure 2:**
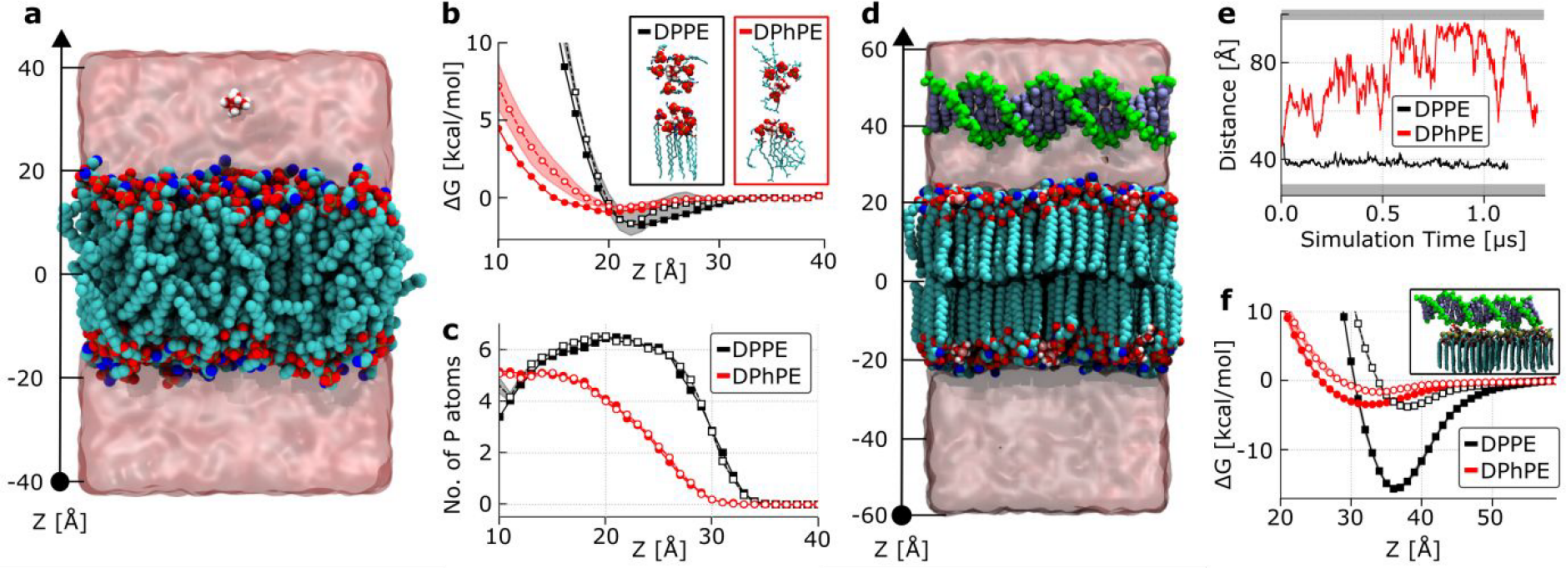
MD simulation of Mg^2+^-modulated binding of DNA to fluid and gel-phase membranes. (a) Typical system used for the replica-exchange umbrella sampling simulations of Mg^2+^ affinity to a lipid membrane. Non-hydrogen atoms of the lipid (DPhPE) membrane are shown as blue (N), tan (P), red (O), and cyan (C) spheres. One magnesium ion and its first solvation shell, Mg[H_2_O]_6_^2+^, are shown explicitly by using red and white spheres; the semitransparent surface illustrates the volume occupied by the MgCl_2_ solution. (b) Free energy of Mg[H_2_O]_6_^2+^ versus distance to the midplane of the lipid membrane. The *z*-axis is defined in panel (a). Insets illustrate a representative coordination of the magnesium ions by lipid headgroups at the minimum of the respective free energy curves. Open and filled symbols indicate data for single ion and 100 mM MgCl_2_ buffer conditions, respectively. (c) Number of phosphorus atoms of the lipid headgroups within 1 nm of a Mg[H_2_O]_6_^2+^ ion versus its distance from the membrane midplane. (d) Typical system used in simulations probing dsDNA affinity to a lipid membrane; DPPE membrane is shown. The backbone and bases of the 21-base pair DNA fragment are shown in green and blue, respectively. The DNA’s backbone is connected to itself across the periodic boundary of the system. (e) Distance between the center of the DNA and the midplane of the lipid membrane during free-equilibration simulations. The shaded region at the bottom of the plot shows the approximate location of the lipid membrane with which the DNA is interacting, while the top shaded region marks its periodic image along the *z*-axis. (f) Free energy of the 21 bp DNA fragment versus its distance to the membrane midplane. The inset image illustrates a representative instantaneous configuration of Mg[H_2_O]_6_^2+^ ions near DNA at its free energy minimum. Open and filled symbols indicate data for 4 and 20 mM concentration of MgCl_2_, respectively.

To quantitatively examine the affinity that Mg^2+^ ions display to fluid-phase and gel-phase membranes, we used the replica-exchange umbrella sampling (REUS) method^53^ and determined the potential of mean force (PMF) between one Mg^2+^ ion and either a DPhPE or a DPPE membrane, as illustrated in Figure 2a. PE lipids, similarly zwitterionic, and differing only slightly in chemistry of the headgroup from PC lipids, were selected for these quantitative simulations because of the possibility of accurately describing the interactions between the PE headgroup and Mg^2+^ by using a magnesium hexahydrate (Mg[H_2_O]_6_^2+^) model^54^ and the CUFIX corrections^55^ to the interactions between Mg[H_2_O]_6_^2+^, the phosphate and amine groups of the PE lipid,^56^ and the phosphate groups of the DNA.^57^ The resulting PMF curves, presented in Figure 2b, show a small yet clearly discernible difference between the binding affinities of Mg^2+^ for gel (DPPE) and liquid (DPhPE) membranes: the PMF minimum near the PE headgroups is 1.0 kcal/mol lower for the gel-phase membrane than for the fluid-phase one. The difference is found to originate from a differential coordination of Mg^2+^ ions by the lipid headgroups, illustrated by plots in Figure 2c. Indeed, representative configurations such as those shown in the insets of Figure 2b demonstrate that at the PMF’s minima approximately four phosphate groups surround each Mg^2+^ ion for the fluid phase membrane, while this number increases to ∼6 for the gel phase. To verify that this result holds at lower Mg^2+^ concentrations, we repeated our PMF calculations for a system containing a single Mg^2+^ ion. The resulting PMF curves largely overlapped with the higher Mg^2+^ concentration data, as shown in Figure 2b,c.

Having confirmed the preferential affinity of Mg^2+^ to gel-phase bilayers, we proceeded to directly probe the effect of lipid phase and magnesium on DNA–membrane interactions. To do so, we included a dsDNA fragment in our all-atom systems, placing it parallel to the surface of either a fluid-phase (DPhPE) or a gel-phase (DPPE) membrane, as shown in Figure 2d. By connecting the DNA molecule to itself over the periodic boundary of the simulation box, we ensured the duplex remained parallel to the lipid membrane over the course of MD simulations. To represent solvent conditions corresponding to a low (20 mM) concentration of bulk MgCl_2_, we calculated the number of Mg^2+^ ions bound to each leaflet of the DPhPE (5 ions) and DPPE (16 ions) bilayers using the single ion PMFs (Figure 2b) and the Langmuir isotherm model.^58^ During the microsecond free-equilibration simulations, the DNA was observed to transiently bind to the surface of the DPhPE membrane and remain permanently bound to that of DPPE, as illustrated by the plots in Figure 2e as well as Movie S1 and Movie S2, clearly indicating a difference in the binding affinity. Importantly, Mg^2+^ ions were found to sustain bridging interactions between DNA and the lipid headgroup when DNA approached the membrane, presented in the inset to Figure 2f, as well as in Movie S3 and Movie S4.

To quantify the difference in the binding affinity of dsDNA to the membranes, we used the REUS method to determine the PMF between the DNA fragment and lipids. To increase convergence of the PMF calculations, we constrained Mg^2+^ ions to remain within close proximity of the headgroups of the lipid bilayers. The resulting PMFs, presented in Figure 2f, show a pronounced (over 10 kcal/mol) effect of the lipid phase on the depth of the PMF minimum, which is consistent with the DNA binding behavior observed in free equilibration simulation (Figure 2e). Much shallower PMF minima were observed when the REUS simulations were repeated in the absence of Mg^2+^ ions, at 150 mM NaCl (Figure S13). To determine the effect of Mg^2+^ concentration, we repeated the PMF calculations for even lower (4 mM) concentration of bulk MgCl_2_ (Figure 2f). While the magnitude of the attractive interactions reduced by severalfold, the gel phase membrane retained a much stronger affinity for dsDNA than the fluid-phase one. Note that although the difference in the depth of the PMF minima is expected to accurately report on the difference in the DNA binding energy corresponding to membrane and ion conditions, the absolute value of the PMF minimum cannot be used to extract an accurate *K*_D_ value because the PMFs were obtained with the DNA arranged parallel to the membrane and thus do not account for entropic terms associated with DNA reorientation.

The insights gained from our simulations, summarized in Figure 2, robustly confirm the experimental observations and elucidate the molecular details of the role that divalent cations play in the interactions between DNA molecules and lipid bilayers. Even though divalent cations can bind to zwitterionic lipids in both liquid and gel phases, as also indicated by ξ-potential measurements, their affinity for gel-phase membranes is higher due to the increased number of lipid headgroups coordinating the Mg^2+^ ion. Simulations indicate that the latter causes a stronger attractive interaction of dsDNA with gel-phased bilayers, in excellent agreement with our experimental data. The results also confirm that the attachment emerges due to Mg^2+^-mediated bridging between the negatively charged phosphates in the lipid headgroups and the DNA backbone.

### Cation *Screening* Regulates Membrane Attachment of Amphiphilic DNA Constructs

In view of the negative surface charge displayed by zwitterionic PC bilayers (Supporting Information Discussion 2), we hypothesize that while cations do not facilitate DNA–lipid bridging in liquid-phase bilayers, they still play a regulatory role modifying electrostatic interactions between the two molecules and thus modulate DNA–lipid complexation caused by another attractive force. Even if cations are not *driving* the attachment, their charge can still *enable* it.

Hydrophobic modifications on DNA nanostructures are known to result in a strong affinity for lipid bilayers regardless of their phase.^59,60^ Therefore, to precisely assess and exploit the modulating effect of charge concentration on the DNA–lipid interactions, we equipped our DNA duplexes with cholesterol moieties and studied their adhesion to GUVs as modulated by ionic strength.

**Figure 3:**
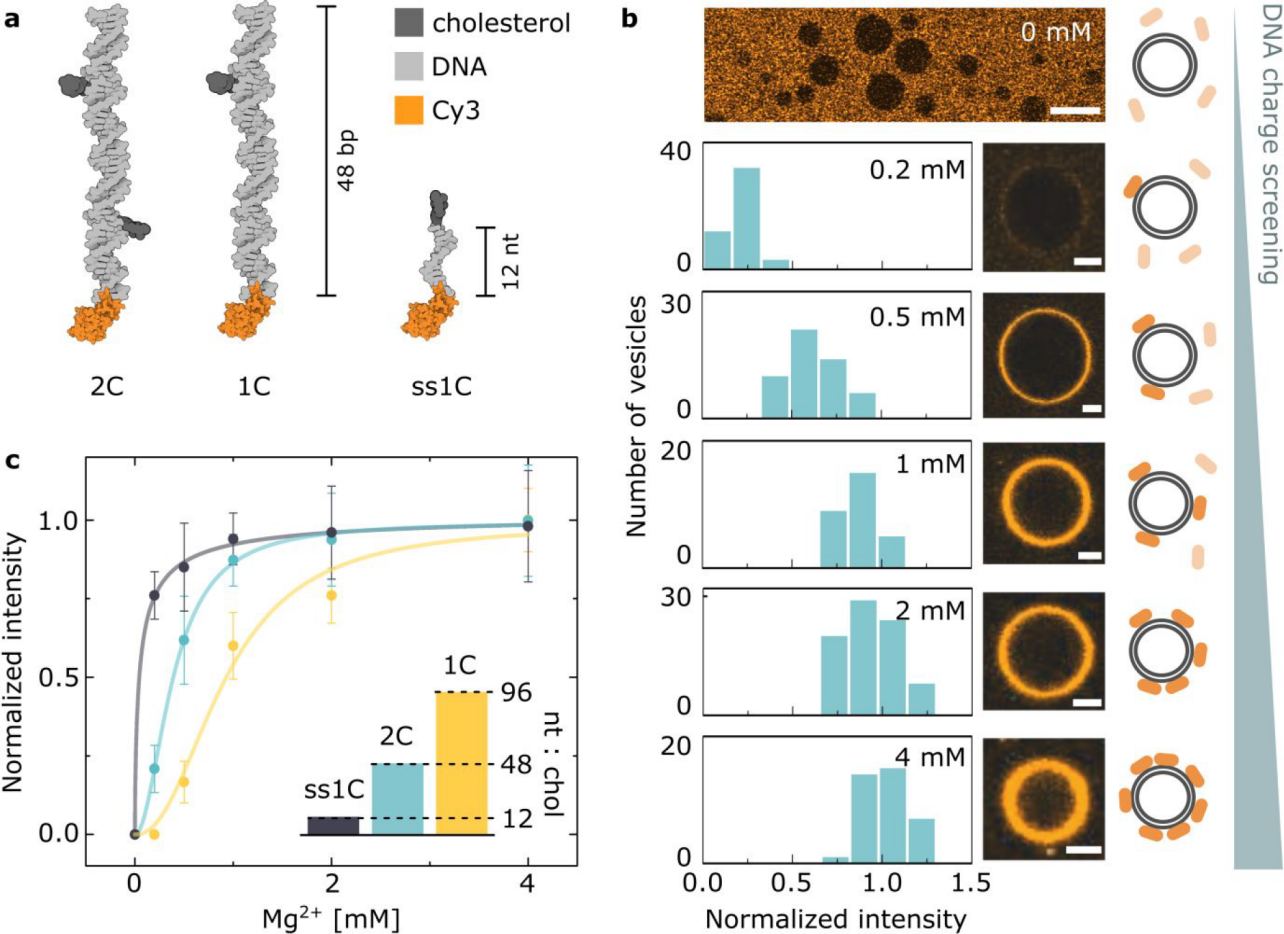
Cations modulate the affinity of hydrophobically modified DNA to liquid-phase PC bilayers. (a) A sketch illustrating the designed library of DNA constructs varying in nt:chol ratio (details of the designs can be found in Section S1, while the oligonucleotides’ sequences can be found in Table S1). (b) Fluorescence–intensity distributions of the Cy3-labeled 2C DNA constructs as recorded on the surface of POPC GUVs at varying Mg^2+^ concentration and representative confocal micrographs. Scale bars: 5 μm. The top image is recorded in the absence of magnesium, where no DNA accumulation on the GUVs was detected. Scale bar: 20 μm. The contrast of the top image was increased for clarity, while no contrast adjustments are applied to the micrographs below it. (c) Magnesium dependency of the peak of the intensity distributions in panel (b) along with analogous data recorded with 1C and ss1C structures (Figure S18 and Table S3). The bar plot in the inset shows the differences in the ratio between the number of nucleotides and cholesterol tags. The solid lines are best fits to a Hill function, used only for illustrating the observed trend (Section S3 and Table S2).

The construct we first consider in this section is a DNA duplex (Figure 3a and Table S1) tagged with two cholesterol molecules (2C) as reported previously.^13^ Its ability to decorate the surface of POPC GUVs (L_d_ phase) was studied at room temperature for a range of magnesium concentrations between 0 and 4 mM, spanning the physiologically relevant values for serum (0.75–1.25 mM^61^). The stability of the construct was not significantly affected by changes in cation concentration, as shown with PAGE (Figure S14) and UV–vis absorbance spectrophotometry (Figure S15). After incubating the Cy3-labeled structures with GUVs, the fluorescence intensity of the DNA membrane coating was measured, as summarized in Figure 3b. A strong dependency of the degree of DNA adsorption on magnesium concentration is readily observed, with denser DNA coatings found for higher salt concentrations and a lack of any detectable attachment observed in the absence of salt.

These results directly confirm the presence of electrostatic repulsion between the DNA and the lipid headgroups, which can be screened by increasing concentrations of cations. We can thus argue that the interactions between amphiphilic DNA constructs and lipid bilayers are regulated by two competing effects: the attractive hydrophobic force between the cholesterol moieties and the bilayer core and the electrostatic repulsion between the lipid headgroups and the DNA motifs. One can therefore classify different hydrophobically modified DNA constructs using a “tug-of-war” ratio between the number of negatively charged nucleotides and that of cholesterol moieties (nt:chol). Constructs differing in this measure should exhibit different degrees of membrane affinity dependent on ion concentration, given that cations will screen electrostatic repulsion without affecting the cholesterol–lipid attraction.

To test this hypothesis, we introduced two additional structures, both modified with a single cholesterol molecule: a 48 bp DNA duplex similar to the one described above (1C) and a 12 nt single-stranded DNA (ss1C). Figure 3c presents the fluorescence intensity of the DNA-coated vesicles as a function of magnesium concentration for these three structures, together with the inset bar chart visualizing their nt:chol: 96 (1C), 48 (2C), and 12 (ss1C). We observed that for low nt:chol ratios the amount of screening required to achieve a given degree of attachment is lower.

In addition, we confirmed that the DNA attachment can also be facilitated by calcium (Figure S16) and potassium ions (Figure S17); however, at least an order of magnitude higher concentration of monovalent ions was required to match the trends observed with divalent ones. The evidence that monovalent cations also enable attachment supports our interpretation that the modulating effect of cations for the interactions between cholesterol-modified nanostructures and lipid membranes is indeed a result of charge screening: a process that occurs regardless of cation valency, albeit more efficiently for higher valency. Conversely, as discussed above (Figure 1), the attractive interaction between unmodified DNA constructs and gel-phase PC membranes results from bridging, and therefore it emerges only with divalent cations, as monovalent ones are unable to bridge.^42^ In Supporting Information Discussion 3 we provide a detailed comparison between the screening abilities of the four major cation species present in biological systems (sodium, potassium, calcium, and magnesium), included at concentrations relevant to key biological environments. The observed trends could form the basis of cation-responsive nanodevices acting as ionic strength sensors^62^ or constructs with functionalities activated only at certain cellular locations.^63,64^

PAGE analysis performed in the presence of various Mg^2+^ concentrations further illustrates the profound effect of screening on the DNA behavior (Supporting Information Discussion 4) and provides an additional confirmation of constructs’ stability throughout the experiment. Similarly, note that measurements of salt-dependent membrane affinity performed with ss1C construct also constitute a control ruling out the possibility of instability-related artifacts, since ss1C is a ssDNA molecule to which both the fluorophore and the cholesterol modification are covalently linked.

Finally, note that cations could also help screening DNA–DNA repulsion, hence facilitating the formation of denser DNA coatings.^27,65^ However, this effect alone is unlikely to account for the trends observed in Figure 3, since if DNA–DNA repulsion was the key factor hampering membrane attachment, one would still observe a degree of coating in total absence of cations.

### Cation-Regulated Activation of a Membrane-Bound DNA Nanomachine

**Figure 4:**
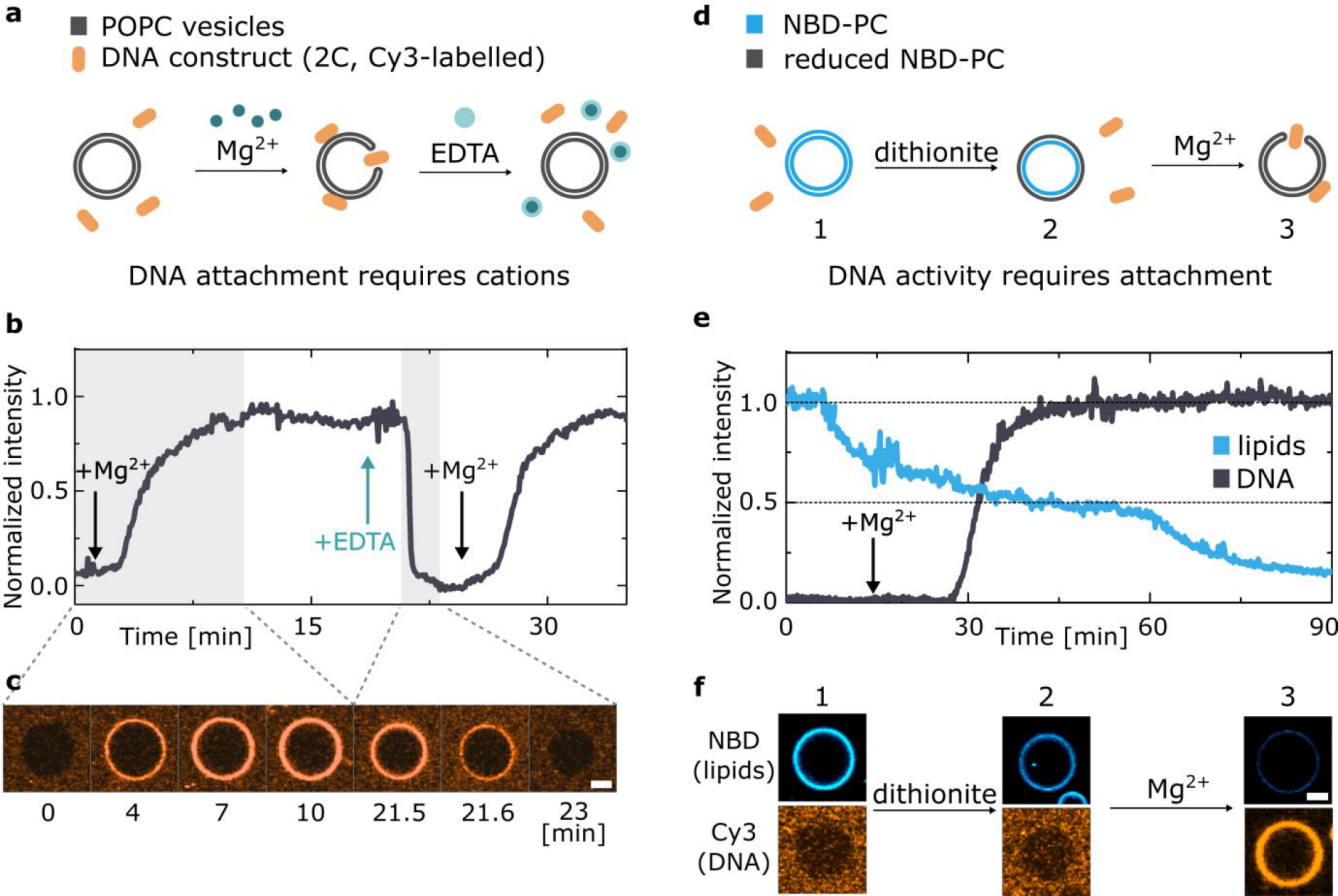
Cation-regulated reversible DNA–membrane binding and activation of a synthetic enzyme. (a) Schematic representation of the mechanism leading to reversible DNA–membrane attachment upon addition of magnesium and its removal by means of chelating agent EDTA. (b) Representative fluorescence intensity trace of Cy3-labeled DNA nanostructures (2C) as recorded from POPC GUVs. DNA attachment and detachment are triggered by the addition of magnesium chloride and EDTA, respectively, as indicated by arrows. Delays associated with the diffusion of added Mg^2+^ and EDTA through the experimental chamber result in short lag times before changes in fluorescence are observed (for details see Section S3). (c) Confocal micrographs from the highlighted gray areas of the trace in (b), demonstrating the attachment and detachment transients. Scale bar: 5 μm. (d) Schematics of the NBD–dithionite reduction assay used to demonstrate cation-activated lipid scrambling. Upon addition of dithionite only the outer leaflet of NBD-tagged membrane is bleached. Magnesium addition induces the insertion of 2C DNA, which creates toroidal membrane pore and induces interleaflet mixing, leading to further fluorescence decrease. (e) Representative trace of the fluorescent intensity of NBD-labeled lipids (blue) upon addition of dithionite, alongside the trace representing Cy3-labeled DNA coating of the vesicle (black) appearing after addition of magnesium (arrow). See Figure S19 for a noninserting control and Figure S20 for an additional example. (f) Representative confocal micrographs, showing the fluorescence of both DNA and lipids at each stage of the experiment described in (d). Scale bar: 5 μm.

The dependence of the membrane affinity of hydrophobically tagged DNA on salt concentration offers a route to reversibly trigger attachment and detachment by adding and removing cations. As summarized in Figure 4a–c, GUVs were incubated with 2C DNA nanostructures, initially in the absence of cations. Magnesium was then added, triggering the attachment of DNA. The subsequent addition of EDTA, chelating the magnesium ions, produced a decrease in fluorescence to background levels. Finally, adding further free magnesium caused the DNA to bind the membranes once again, demonstrating full reversibility of the salt-regulated attachment process.

The reversible effect of cations on the membrane attachment of cholesterol-modified DNA nanostructures is reminiscent of the cation-dependent activity seen in a number of natural transmembrane proteins.^66,67^ Inspired by their biological analogues, we demonstrated that the activity of synthetic DNA nanodevices can also be regulated by cations. To this end, we consider the functionality of 2C DNA constructs, which upon membrane insertion form toroidal pores in lipid bilayers, triggering the exchange of lipids between the inner and outer leaflets, similar to scramblase enzymes.^12,13^ Here we report that the activity of such a synthetic enzyme can be triggered with cations, remarkably alike the natural scramblases.^66^

We use a previously described assay^12,13,68,69^ based on the reduction of NBD, a dye that while fluorescent in its oxidized state readily bleaches upon exposure to a strong reducing agent. As summarized in Figure 4d–f, we prepared GUVs in which both leaflets contained NBD-labeled lipids. The vesicles were initially incubated with the 2C DNA in the absence of ions, which as expected did not attach onto the membranes, and were therefore in an inactive state. We then added the reducing agent dithionite (S_2_O_4_^2–^) in the outer solution, which being unable to penetrate the GUVs caused bleaching only of the NBD molecules on the outer bilayer leaflet, but not of those on the inner leaflet, resulting in ∼50% loss of fluorescence. The addition of magnesium at this point activated the nanostructures, causing their insertion into the membranes. The functional synthetic enzymes enabled mixing of the inner and outer membrane leaflets, and the exposure of previously unbleached NBD fluorophores to the reducing agent, causing a decrease of the NBD emission below the initial 50%.

As a control, the experiment was repeated with 1C DNA, which can bind to the membranes upon the addition of magnesium but does not create a toroidal pore like 2C does (Figure S19). No significant decrease of the NBD emission below the initial bleaching of the outer leaflet was observed, further confirming that the behavior detected with 2C is indeed attributed to the DNA-induced lipid scrambling and that the addition of magnesium acted as an external stimulus for activating the enzyme.

## Conclusions

In summary, we have explored the mechanisms through which the interactions between dsDNA and zwitterionic bilayers can be regulated by the action of cations, offering a number of new routes for modulating DNA–membrane interplay in response to changes in system parameters and the application of external stimuli.

First, we reported on the emergence of attractive forces between DNA and bilayers, a phenomenon which occurs only for gel-phase membranes in the presence of divalent cations and is absent for liquid-phase bilayers or if only monovalent salts are included. We ascribe the observed adhesion to cation-mediated bridging facilitated by the affinity of divalent cations to the headgroups of gel-phase PC bilayers, as confirmed by all-atom MD simulations.

Second, we examined the screening effect of cations, studying the interactions between liquid-phase PC bilayers and DNA nanostructures modified with cholesterol moieties. Despite the presence of the highly hydrophobic tags, we showed that the Coulomb repulsion between liquid membranes and the negatively charged DNA cannot be overcome by cholesterol modifications only; cations are required to screen electrostatic forces and through that enable membrane attachment.

These electrostatic phenomena are key to a complete understanding of the complex DNA–lipid interactions in biology, where a vast number of processes take place at the membrane interface. Virus cell entry^70^ and RNA transport from the nucleus^71^ are just two examples. Moreover, our observations have implications beyond DNA–lipid interactions, as analogous processes could take place also for other charged biomolecules. For example, we speculate that the changes in protein charge and stability^72^ may influence the proteins’ electrostatic interactions with membranes and play a role in disease pathways, as observed for instance with amyloid fibrils in neurodegenerative conditions such as Alzheimer’s and Parkinson diseases.^73−75^ Our findings have numerous implications for natural systems, yet the benefits of our work are most prominent in the field of biology-inspired nanoengineering.

Here, we focused on demonstrating how the two electrostatic phenomena, bridging and screening, can be exploited by DNA nanotechnology to program the reversible membrane attachment of functional nanostructures. We introduced changes in cation bridging-driven membrane attachment by tuning independent physicochemical parameters, including temperature and sterol content, that cause phase transitions in the membranes. Exploring the effect of screening, we showed that the membrane affinity of amphiphilic DNA constructs varies with salt concentration depending on their charge-to-hydrophobicity ratio. The latter parameter is especially significant in designing biomedical DNA nanostructures functional in physiological conditions, where ionic composition is regulated within well-defined ranges.^76,77^ We illustrated the importance of the charge-to-hydrophobicity ratio by designing a library of DNA duplex constructs with tunable responses to changes in ion concentration.

Our findings will be instrumental to informing the design of biomimetic DNA-based nanodevices. Hydrophobically modified and unmodified DNA nanostructures have been proposed as vectors for the intracellular delivery of drugs and genetic material,^78,79^ and we argue that a precise control over their interactions with biological membranes in physiological ionic conditions could be key to the optimization of their performance. The same logic applies to DNA nanodevices used for probing biological phenomena *in vivo* and *in vitro*, such as those designed to measure cell-exerted forces,^80^ study the interactions between membrane proteins,^81^ and perform super-resolution optical imaging.^82^ Even though the studies presented here feature minimalistic DNA probes, they illustrate universal electrostatic phenomena that will affect more complex nanostructures as well. Although the dependency of these interactions on the design parameters (like lipid-facing surface area and stiffness) are yet to be assessed, cation effects are of high importance for membrane-binding DNA origami platforms^9,83^ as well as constructs internalized by cells.^84,85^

As a first example of how the modulating effect of cations can be exploited to rationally design new and responsive biomimetic devices, we present a magnesium-dependent synthetic scramblase enzyme. One can envisage a range of opportunities becoming available to more complex architectures, with DNA-based membrane channels that change their shape, orientation, and activity, similar to natural membrane proteins they are designed to mimic.

## Supporting Information Available

Experimental and computational details, DNA sequences, nanostructures’ and membranes’ characterization, supporting experiments (PDF)
Movies S1–S4 descriptions (PDF)
Movie S1 (AVI)
Movie S2 (AVI)
Movie S3 (AVI)
Movie S4 (AVI)

## Supplementary Material

ja1c00166_si_002.pdf

ja1c00166_si_003.avi

ja1c00166_si_004.avi

ja1c00166_si_005.avi

ja1c00166_si_006.avi
