# Supplementary material for: Cations Regulate Membrane Attachment and Functionality of DNA Nanostructures
Source: J Am Chem Soc. 2021 May 7;143(19):7358–67. doi: 10.1021/jacs.1c00166 (PMC8154537; doi:10.1021/jacs.1c00166)
Supplement: Supplementary file 1 — ja1c00166_si_001.pdf [file ja1c00166_si_001.pdf]

Supporting information for

Cations regulate membrane-attachment and functionality of DNA nanostructures.

Diana Morzy[1,♦], Roger Rubio-Sánchez[1,♦], Himanshu Joshi[2,♦], Aleksei Aksimentiev[2,3,*], Lorenzo Di Michele[4,1,*], Ulrich F. Keyser[1,*]

[1] Cavendish Laboratory, University of Cambridge, JJ Thomson Avenue, Cambridge, CB3 0HE, United Kingdom

[2] Department of Physics, University of Illinois at Urbana−Champaign, 1110 West Green Street, Urbana, Illinois 61801, United States

[3] Beckman Institute for Advanced Science and Technology, University of Illinois at Urbana-Champaign, 405 North Mathews Avenue, Urbana, Illinois 61801, United States

[4] Department of Chemistry, Molecular Sciences Research Hub, Imperial College London, London W12 0BZ, United Kingdom

♦ These authors contributed to the work equally

* Corresponding authors: (UFK) ufk20@cam.ac.uk , (LDM) l.di-michele@imperial.ac.uk, (AA) aksiment@illinois.edu

Contents

List of figures. 2
List of tables 2
S1 DNA nanostructure assembly 3
S2 Vesicle preparation 3
S3 Confocal microscopy imaging 3
S4 DLS and zeta potential 5
S5 Assessment of DNA constructs' temperature stability using UV-Vis absorption spectroscopy 6
S6 Native polyacrylamide gel electrophoresis (PAGE) 6
S7 Dithionite quenching of NBD-lipids 7
S8 Differential Scanning Calorimetry of DPPC vesicles 7
S9 Molecular Dynamics Methods 7
S10 Supplementary Discussions 10
S11 Figures 17
S12 Tables 38
References 39

List of figures

Supplementary Figure D1.1 10

Supplementary Figure D2.1 13
Supplementary Figure D2.2 13

Supplementary Figure D3.1 15

Supplementary Figure D4.1 16
Supplementary Figure D4.2 17

Supplementary Figure 1. 18
Supplementary Figure 2. 19
Supplementary Figure 3. 20
Supplementary Figure 4. 21
Supplementary Figure 5. 22
Supplementary Figure 6. 23
Supplementary Figure 7. 24
Supplementary Figure 8. 25
Supplementary Figure 9. 26
Supplementary Figure 10. 27
Supplementary Figure 11. 28
Supplementary Figure 12. 29
Supplementary Figure 13. 30
Supplementary Figure 14. 31
Supplementary Figure 15. 32
Supplementary Figure 16. 33
Supplementary Figure 17. 34
Supplementary Figure 18. 35
Supplementary Figure 19. 36
Supplementary Figure 20. 37

List of tables

Table 1 38
Table 2 38
Table 3 38
Table 4 38

S1 DNA nanostructure assembly

All the reagents used in this work were acquired from Sigma Aldrich, unless stated otherwise. Each single-stranded DNA oligo was analysed using the NUPACK suite[1], in order to prevent formation of secondary structures, and to ensure sufficient yield of folding. Oligonucleotides were obtained from Integrated DNA Technologies, Inc. All the strands were dissolved to a final concentration of 100 µM: unmodified ones in IDTE buffer (10 mM Tris, 0.1 mM EDTA (Ethylenediaminetetraacetic acid), pH 8.0) and labelled ones in Milli-Q purified water. Strands were then stored at 4°C, except for dye-modified ones, which were stored in -20 °C.

In order to fold the designed structures, the strands were mixed to a final concentration of 1 µM in TE buffer (10 mM Tris, 1 mM EDTA, pH 8.0), with cholesterol-modified strands heated beforehand to 70 °C for 10 min. In the case of UV-vis measurements and PAGE analysis, the structures were folded in a buffer with magnesium concentration stated for each experiment. DNA duplexes were left for half an hour at room temperature before proceeding with experiments. Folded structures were all stored at 4°C. The structures used in this work were:

- 36 bp-long duplex: D1' + D1'', Cy3-labelled
- 48 bp-long duplex: (D1 + D1') + (D2+D2'), Cy3-labelled, with 0/1/2 cholesterol modifications

The sequences and details of modifications are listed in Table 1, as well as illustrated in Supplementary Fig. 1.

S2 Vesicle preparation

GUVs used in the assay were prepared with electroformation, as reported previously[2]. POPC (1-palmitoyl-2-oleoyl-glycero-3-phosphocholine) or DPPC (1,2-dipalmitoyl-sn-glycero-3-phosphocholine) with an addition of NBD-PC lipids (1-palmitoyl-2-{6-[(7-nitro-2-1,3-benzoxadiazol-4-yl)amino]hexanoyl}-sn-glycero-3-phosphocholine), all acquired from Avanti® Polar Lipids, were used in a ratio of 200:1, with the final concentration of 5 mg/ml in chloroform. 600 µl of 1 M sorbitol in 200 mM sucrose was used as a buffer. The osmolality of the buffer was around 1200 mOsm, with all the dilution buffers used in the experiments adjusted accordingly. Since for cell plasma the osmolality ranges between 275 - 325 mOsm[3], therefore we do not claim a biological osmolality. All the buffers were adjusted to pH 7.5 (using sodium hydroxide and hydrochloride solutions) - the value within the acidity range observed in natural systems[4]. In the case of electroformation of GUVs containing lipids with high-melting temperatures (i.e. DPPC), the process was performed at 60°C to ensure bilayer fluidity.

LUVs used for the zeta (ξ) potential measurements were prepared with extrusion, using a commercially available extruder (Avanti® Polar Lipids, Avanti Mini Extruder), following producer's protocol. Whatman® Filter Supports and Whatman® Nuclepore Track-Etched Membranes (200 nm) were obtained from Sigma Aldrich. The DPPC layer was hydrated in 200 mM sucrose, and the extrusion was performed at 60°C to ensure bilayer fluidity.

S3 Confocal microscopy imaging

Confocal microscopy images were acquired on an Olympus FluoView filter-based FV1200F-IX83 laser scanning microscope using a 60x oil immersion objective (UPLSAPO60XO/1.35). NBD excitation was performed using a 25 mW 473 nm laser diode at 1% laser power, with emission collected between 490 and 525 nm. Cy3 excitation was performed using a 1.5 mW 543 nm HeNe laser at 3% laser power, with emission collected between 560

and 590 nm. FIJI was used to analyse the images[5]. In the experiments listed below 20 µl of electroformed liposomes were incubated for 1 h with 50 µl of DNA structures diluted in an osmotically balanced glucose-based buffer, also containing 25 mM HEPES (4-(2-hydroxyethyl)-1-piperazineethanesulfonic acid). The difference in sugar densities caused sucrose-filled vesicles to sediment to the bottom of the incubation chamber.

The experiment inducing attachment/detachment of nanostructures to the membrane (described in Fig. 4 of the main text) was performed with a 48 bp-long 2C (2 x cholesterol) duplex. Initially, no Mg^{2+} was present in the chamber; it was subsequently added after recording a number of frames, to a final concentration of 4 mM, without disrupting acquisition. Upon observing DNA attachment, EDTA was added to the chamber to a final concentration of 10 mM. The next Mg^{2+} addition increased its concentration to 20 mM. All of the added substances were diluted in osmotically balanced glucose buffer. Images were recorded every 10 s, with a sampling speed of 2.0 µs/pixel, and FIJI[5] was used to measure changing intensity of the DNA vesicle coating.

In order to avoid GUV disruption or drift, the Mg/EDTA solutions are gently added to the surface of the chamber, without active mixing. Therefore, the changes in the attachment are only detected after the ions/chelating agents have diffused to the observed part of the chamber. As the diffusion rate for Mg^{2+} at room temperature (≈ 25 °C) is reported to be in a range of [100, 1000] $\mu m^2/s$ [6], we do expect a delay on a minute scale in the diffusion on millimeters distances.

FRAP measurements were performed to confirm the presence of gel and liquid phases in the experiments, the results are collected in Supplementary Fig. 6. Using the FRAP function of the microscope's software (tornado mode), a spot of Ø4.4 µm was bleached and the fluorescence recovery observed. 10 frames were collected pre-bleaching. Bleaching was performed over 0.5 s with 99% laser power and the fluorescence recovery was recorded for 100 frames.

Collected recovery curves were fitted using exponential function (1).

$$I_t = A\left(1 - \exp\left(-\frac{t}{\tau}\right)\right) + I \tag{1}$$

where I_t – fluorescence intensity in time t, A – fitting parameter, I – final intensity after recovery, τ - recovery time constant

τ was then used to calculate recovery half-time as in (2)

$$t_{\frac{1}{2}} = \tau ln2 \tag{2}$$

Which in turn enabled obtaining diffusion coefficient D following the formula (3)

$$D = \frac{0.88r^2}{4t_{\frac{1}{2}}} \tag{3}$$

where r – radius of bleached area.[7]

For the experiments presented on Fig. 3, series of representative images from across the sample were collected

for 2C, 1C and ss1C (D1 in Tab. S1) structures, incubated in magnesium concentration varying from 0 to 4 mM. The intensities of vesicles were measured, and an average value (Gaussian fit to histograms) for every condition was obtained and presented in Table S2, as well as on the plot in Fig. 3b. The error bars represent Gaussian RMS width. Data points were fitted to a Hill function (4):

$$I = \frac{I_{max}[c]^n}{K^n + [c]^n} \tag{4}$$

where I – fluorescence intensity, I_{max} – maximum intensity observed, $[c]$ – ion concentration, n – Hill coefficient, K – ion concentration associated with 50% of maximum attachment $(I = 0.5)$

The dissociation constants were then calculated using equation (5)

$$K_d = K^n \tag{5}$$

The parameters obtained for each studied structure are collated in Table S3. We have opted for the Hill function in order to describe empirically ion-nucleic acid interactions, as done previously[8–10]. However, it must be noted that the equation was employed here to allow an illustrative comparison of studied constructs, rather than as a mathematical model of the underlying process. Therefore, the calculated parameters do not necessarily carry the meaning most frequently ascribed to them.

In the case of temperature ramps presented in Fig. 1, borosilicate glass capillaries (CM Scientific, with internal section of 2 mm × 0.2 mm) were cleaned by sonication cycles of 15 min (Hellmanax III [purchased from HellmaAnalytics] at 2%/Isopropanol/milli-Q water). Once dried, they were passivated by filling them with a solution of bovine serum albumin (BSA) 0.1% and incubated at 60°C for one hour. They were subsequently cleaned with water to remove excess of BSA, and filled with vesicle samples. The sides were sealed using a two component epoxy-resin hardener onto a clean glass cover slip. The coverslip was fixed to a custom-built copper plate connected to a Peltier element using aluminium tape and coupled to a thermocouple for temperature control. Micrographs of these systems were acquired using a Leica TCS SP5 confocal microscope equipped with an HC PL APO CORR CS 40× / 0.85 dry objective. NBD (excitation maximum – 490 nm; emission maximum – 525 nm) and Cy3 (excitation maximum – 550; emission maximum – 570 nm) fluorophores were tracked using an Ar-ion (488 nm) laser and a He-Ne (543 nm) laser lines, respectively. Data points were collected every 5 °C between 20 and 40 °C, and every 1 °C between 40 and 50 °C. The sample was equilibrated for a minute upon each temperature change. To assess the kinetics of the DNA desorption upon phase transition, the same experimental system was used to instantaneously cross the T_m of DPPC (increase from 35 °C to 50 °C), while imaging.

S4 DLS and zeta potential

The measurements were performed on a Zetasizer Nano ZSP (Malvern Panalytical) with an excitation wavelength of 633 nm and a scattering angle fixed at 173°. For assessing hydrodynamic radius, 100 µl of 10× diluted vesicles were measured. The ξ-potential measurements were performed in a DTS1070 cell, with [DNA] = 0.1 µM, $[Mg^{2+}]$ = 1 mM, 10× diluted LUVs in 800 µl of degassed 200 mM sucrose solution. 3 measurements were taken for each condition, each consisting of 12 runs.

Lognormal distribution curves were fitted to the obtained histograms of LUVs size, following function (6).

$$y = y_0 + \frac{A}{\sqrt{2\pi}wx} e^{\frac{-\left[ln\frac{x}{x_c}\right]^2}{2w^2}} \tag{6}$$

where y_0 – offset, x_c – center, w – log standard deviation, A – area.

The standard deviation was calculated using formula (7). Both formulae, reported by Origin software, were used for plotting the data.

$$\Delta y = e^{\ln(x_c)+0.5w^2}\sqrt{e^{w^2} - 1} \tag{7}$$

S5 Assessment of DNA constructs' temperature stability using UV-Vis absorption spectroscopy

The effect of magnesium concentration on the stability of unmodified 48 bp DNA constructs (0C) was assessed using a UV-vis spectrophotometer (Cary 300 Bio, Agilent); thermal studies were performed in order to obtain melting curves of the structures. 100 µl of 1 µM DNA sample folded in the stated concentration of $MgCl_2$ were heated from 10 to 90°C, with a heating rate of 1°C/min. Absorbance spectra were collected at 260 nm, and the melting temperature was obtained from the median of the two linear regions (upper and lower). The data, and their analysis, were processed using Origin software for all measurements taken. Similarly, the melting profiles for the unmodified 36 bp duplex were acquired in quartz cuvettes filled with 1.1 mL of sample, capped with 400 µl of mineral oil and sealed to prevent evaporation. Absorbance measurements were collected over two contiguous temperature ramps: first cooling (90 to 10°C) and then heating (10 to 90°C) at a rate of 0.2°C/min. The melting profile of 36 bp dsDNA is shown in Supplementary Fig. 9, while the melting profiles of 48 bp DNA construct in a range of Mg^{2+} concentration can be found in Supplementary Fig. 15.

S6 Native polyacrylamide gel electrophoresis (PAGE)

Polyacrylamide gel electrophoresis was used to confirm the proper folding of DNA designs. The gels were prepared at a concentration of 10% polyacrylamide, 0.5× TBE (Tris, borate, EDTA) and with 11 mM $MgCl_2$, unless stated otherwise. Addition of 0.01 vol% ammonium persulfate (APS) (10%) and 6.7 × 10^{-4} % N,N,N',N' Tetramethylethylenediamine (TEMED) were used to initialise polymerisation, which proceeded for an hour. Subsequently, 2 µl of a DNA sample was mixed with 0.4 µl of 6× loading dye (15% Ficoll R400, 0.9 % Orange G diluted in Mili-Q water), and then 2 µl of sample were loaded into the well. GeneRuler Low Range ladder (Thermo Fisher Scientific Inc.) was used as a reference. The gel was run in a Mini-PROTEAN R Tetra Cell (Bio-Rad), in 0.5x TBE with 11 mM $MgCl_2$ (unless stated otherwise) at 100 mV for 90 min. Afterwards, the gel was immersed for 10 min in GelRed (Biotium), in order to stain the DNA. Imaging was performed on a GelDoc-It TM (UVP), and FIJI was used to analyse gel images.

The DNA samples were folded in TE buffer with different concentrations of magnesium: 0, 0.2, 0.5, 1, 2, 4 mM. Further experiments were performed with 11, 4, 1 or no magnesium in either the buffer or the gel itself, and

run with DNA folded in respective Mg^{2+} solutions. After running all four gels in the same settings (100 mV for 60 min), the position of bands was compared (Supplementary Discussion 4) to show the effect of charge screening on DNA.

S7 Dithionite quenching of NBD-lipids

The NBD quenching assay described in Fig. 4 was performed with the Olympus FluoView imaging setup described above. Here, the concentration of DNA was 0.11 µM, with no magnesium present in the chamber. Immediately preceding the assay, sodium dithionite was diluted in 1 M Tris (pH 10) to a concentration of 15 mM. This solution was further diluted in osmotically balanced glucose solution, from which 30 µl were added to the chamber. The final concentrations of DNA and dithionite were 0.08 µM and 4.5 mM, respectively. Images were recorded every 10 s, with a sampling speed of 2.0 µs/pixel. After the initial intensity decrease, magnesium chloride, diluted (50 mM) in the osmotically balanced glucose solution, was added to the sample to a final concentration = 4 mM. The imaging continued without interruption. As a control, the same experiment was performed with the 1C structure, which showed similar attachment upon magnesium addition, but no lipid flipping activity occurred (see Supplementary Fig. 19).

S8 Differential Scanning Calorimetry of DPPC vesicles

Differential Scanning Calorimetry (DSC) was performed on DPPC lipid vesicles using standard aluminum DSC pans in a PerkinElmer DSC 4000 instrument. Samples were heated from 20°C to 60°C at 1°C/min, equilibrated at 60°C for two minutes, and then cooled down to 20°C at the same rate. Throughout the ramp, heat flow (mW) was recorded for vesicles in both the presence and absence of dsDNA and Mg^{2+} ions (see Supplementary Fig. 4).

S9 Molecular Dynamics Methods

General simulation protocols. All MD simulations were performed using program NAMD2[11], a 2 fs integration time step, 2-2-6 multiple time stepping, periodic boundary conditions, and particle mesh Ewald (PME) method over a 1-Å resolution grid to calculate the long-range electrostatic interaction[12]. The Nose-Hoover Langevin piston[13] and Langevin thermostat were used to maintain the constant pressure and temperature in the system. An 8-10-12 Å cutoff scheme was used to calculate van der Waals and short-range electrostatic forces. SETTLE algorithm[14] was applied to keep water molecules rigid whereas RATTLE algorithm[15] constrained all other covalent bonds involving hydrogen atoms. CHARMM36 force field parameters described the bonded and non-bonded interactions among the atoms of DNA[16], lipid[17], water and ions[18]. Magnesium ions were modeled as magnesium hexahydrates ($Mg[H_2O]_6{}^{2+}$)[19]. Corrections to non-bonded interactions were applied to improve description of ion-DNA[19], ion-ion, and DNA-lipid interactions[20]. The coordinates of the system were saved every 20 ps. The visualisation, analysis, and post-processing the simulation trajectories were performed using VMD[21] and CPPTRAJ[22].

Initial models of fluid and gel phase membranes. The initial configuration of the 1,2-dipalmitoyl-sn-glycero-3-phosphoethanolamine (DPPE) lipid bilayer membrane containing 64 lipids in each leaflet was generated from the CHARMM-GUI membrane builder[23]. A pre-equilibrated patch of 1,2-diphytanoyl-sn-glycero-3-

phosphoethanolamine (DPhPE) containing 64 lipids in each leaflet was obtained from a previous study[2]. Both membranes were solvated with TIP3P water molecules[24]. Magnesium hexahydrade and chloride ions were placed randomly in the solvent at 300 mM concentration. Fully assembled DPPE and DPhPE membrane systems contained 30,410 and 48,288 atoms, respectively. Each system was first subject to energy minimisation using the conjugate gradient method, which removed steric clashes between the solute and the solvent. The systems were then equilibrated without any restraints for several hundreds of nanosecond in a constant number of atoms (N), pressure (P = 1 bar) and temperature (T = 300 K) ensemble. Anisotropic pressure coupling was used to maintain the constant ratio of the system's dimension within the membrane, allowing the system's dimension normal to the membrane to adjust independently of the other dimensions. Following that, $Mg[H_2O]_6^{2+}$ ions located approximately 30 Å away from the center of the membrane were removed, producing two systems of approximately 100 mM $MgCl_2$ concentration. Each system was then in equilibration for additional 0.5 µs.

PMF of single Mg^{2+} ion. To obtain the PMF of an $Mg[H_2O]_6^{2+}$ ion along the direction normal to the lipid bilayer (*z*-axis), we performed replica-exchange umbrella sampling simulations[25] using the colvar module[26] of NAMD. Starting from the state attained at the end of each 0.5 µs equilibration, a short (3 ns) steered molecular dynamics (SMD) simulation was performed to move one $Mg[H_2O]_6^{2+}$ ion along the *z*-axis from 40 to 10 Å, as measured from the center of the lipid bilayer. Each SMD trajectory was used to create 31 copies of each membrane system differing by the *z*-coordinate of the $Mg[H_2O]_6^{2+}$, one system for each 1 Å sampling window along the *z*-axis. Each replica was simulated for 50 ns having the *z*-coordinate of the Mg atom restrained to the center of the sampling window with the spring constant of 2.5 kcal/mol/ Å^2. During the simulation, the replicas were allowed to exchange the biasing potential between the neighbouring windows with a probability given by the Metropolis algorithm. Finally, we used WHAM[27] to subtract the contribution from the confining harmonic potential and extract the PMF of the $Mg[H_2O]_6^{2+}$ ion along the axis normal to the lipid bilayer membrane. To assess the affinity of $Mg[H_2O]_6^{2+}$ at minimal ionic concentration, we repeated the PMF calculations using a set of simulation systems containing a single $Mg[H_2O]_6^{2+}$ ion. The initial configuration of the 31 replicas (varying in the coordinate of the ion along the *z*-axis from 40 to 10 Å) were obtained by removing all $Mg[H_2O]_6^{2+}$ and chloride ions except one $Mg[H_2O]_6^{2+}$ and two chloride ions from the respective 100 mM of $MgCl_2$ replica systems. Each replica was simulated for approximately 67 ns while restraining the *z*-coordinate of the $Mg[H_2O]_6^{2+}$ ion with respect to the center of the membrane using a force constant of 2.5 kcal/mol/ Å^2. The first 5 ns of the simulation was excluded, and WHAM was used to compute the PMF profile from the later part of the simulation trajectories.

Spontaneous binding of dsDNA to lipid membrane. A 21 base pair fragment of dsDNA was built using the NAB module[28] of AMBERTOOLs. The DNA fragment was made effectively infinite along its helical axis by linking the strands to themselves across the periodic boundary (along the *y*-axis) of the systems. A 10 x 7.1 nm^2 rectangular patch of either DPPE or DPhPE membrane was carved out from the equilibrated systems, matching the systems' dimension along the *y*-axis to that of the DNA. The DNA model was combined with that of each membrane, placing the DNA 5 nm away (along the *z*-axis) from the center of the membrane. The number density, *n*, of ions adsorbed to a membrane patch of area $A_{surface}$ can be computed using the Langmuir isotherm model (8)[29]

$$n = \frac{C_{bulk}}{A_{surface}} \int \overrightarrow{dr}\ e^{-\beta F(\vec{r})} \tag{8}$$

Integrating the free energy profile of the $Mg[H_2O]_6^{2+}$ ions, $F(\vec{r})$, along the bilayer normal over the adsorption region (15 to 25 Å), the model predicts 16 and 5 $Mg[H_2O]_6^{2+}$ ions adsorbed to the 10 x 7.1 nm^2 patch of the

DPPE and DPhPE membrane, respectively, under 20 mM bulk concentration, C_{bulk}, of Mg^{2+}. In our simulation setup, these $Mg[H_2O]_6^{2+}$ ions were uniformly distributed among the lipid head groups in each leaflet of the membrane with their *z*-coordinate set to the minimum of the respective $Mg[H_2O]_6^{2+}$ PMF. These DNA--membrane systems were then solvated with water; sodium and chloride ions were added to neutralize the total charge of the system. The fully assembled systems measured 10 x 7.1 x 14 nm^3 contains 96,329 (DPPE) and 91,542 (DPhPE) atoms. After a brief energy minimisation, the systems were equilibrated without any restraints for 1 µs a constant pressure (P = 1 bar) and temperature (T = 300 K); all system's dimensions were allowed to adjust independently to maintain the target pressure.

PMF of dsDNA. Starting from the microscopic configurations reached after approximately 0.2 µs free equilibration of the respective dsDNA/lipid membrane systems, 41 copies of each system were created using the frames from an 80-ns SMD simulation where the dsDNA's center of mass was moved from 20 to 60 Å along the *z*-axis. In contrast to the free-equilibration simulations, a separate colvars module (distance Z, spring constant 10 kcal/mol/Å^2) was used to ensure the *z*-coordinates of the $MGHH^{2+}$ ions remained at the minima (± 1 Å) of the respective single ion Mg^{2+} PMF; the ions were free to move parallel to the plane of the membrane (*x-y* plane). Two additional systems containing no $MgCl_2$ but 150 mM NaCl were built for each membrane type. Replica exchange umbrella sampling simulations were performed using the 1 Å sampling window for the DNA's center of mass *z*-coordinate which was maintained in each window using a harmonic potential with the spring constant of 2.5 kcal/mol/ Å^2. Each replica was run for approximately 190 ns. The PMFs were reconstructed using WHAM[27]. To compute the PMF profile corresponding to 4mM bulk concentration of $MgCl_2$, we removed all monovalent and divalent ions from the system, except 21 $Mg[H_2O]_6^{2+}$ ions which were required for neutralizing the charge of the DNA backbone. Additionally, we kept 3 and 1 $Mg[H_2O]_6^{2+}$ ions on the each leaflet of the DPPE and DPhPE membrane, respectively, in accordance with numbers prescribed by the Langmuir isotherm model for 4 mM bulk concentration. The initial configurations of the system in 41 sampling windows (varying in the center of mass *z* coordinate of the DNA with respect to the center of the membrane along *z*-axis from 20 to 60 Å) were obtained from the respective 20 mM replica systems. Each sampling window was simulated for 130 ns, WHAM was used to subtract the effect of the harmonic potential and obtain the PMF profile. The first 5 ns of the simulation trajectories were excluded from the WHAM analysis.

S10 Supplementary Discussions

Supplementary Discussion 1. Effects of biologically-relevant cations on DNA-lipid bridging

The presence of high concentration of monovalent cations in cellular fluids has significant implications for the bridging phenomenon observed between gel-phase membranes and non-cholesterolized DNA duplexes, mediated by divalent cations. Since the efficiency of cation-mediated bridging has been shown to depend on monovalent/divalent ion ratio[30], we hypothesized that the observed degree of attachment would vary in different cation mixtures, thus providing another route to fine-tune NA-lipid interactions. In order to exemplify this concept, we have chosen to work with compositions that mimic that of the intercellular media[31], where we introduce 100 mM Na^+ as the monovalent "base" of the solution, and later add different divalent cations at various concentrations (Supplementary Figure D1.1).

As expected, no DNA attachment is observed in the absence of divalent cations. Adding 1 mM Mg^{2+} results in a faint DNA coating, significantly less pronounced than that observed with 1 mM Mg^{2+} in the absence of sodium. This observation confirms that the competition between divalent and monovalent ions weakens bridging, and can be potentially used to modulate DNA-membrane adhesion. At higher, but still biologically-relevant concentrations, magnesium can outcompete sodium, as demonstrated by the strong DNA attachment observed with 20 mM Mg^{2+}.

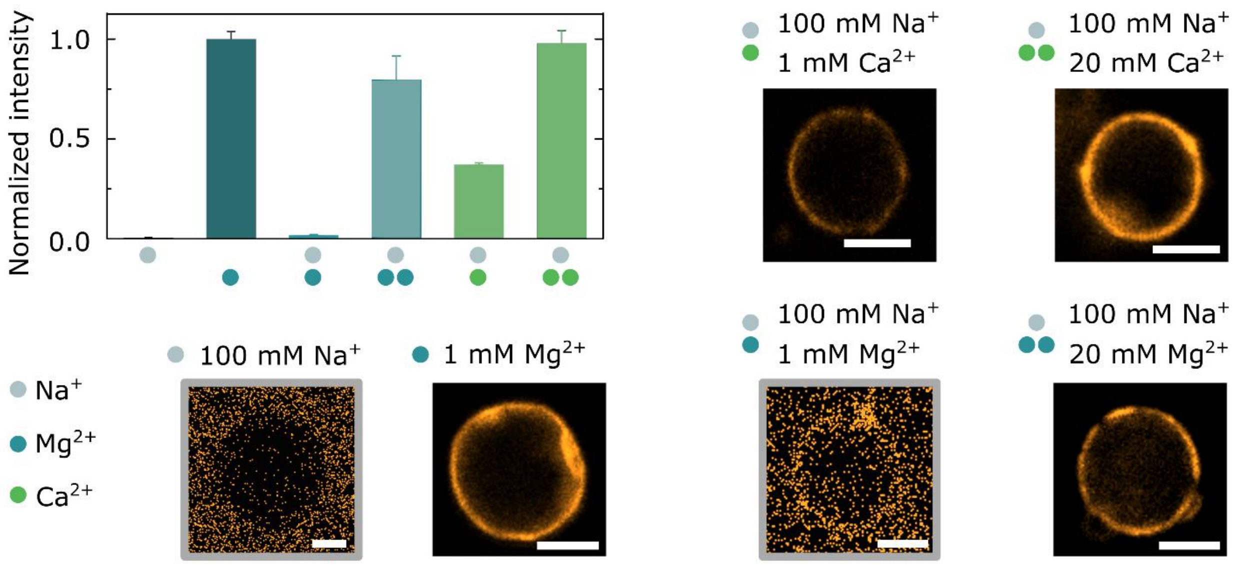

Supplementary Figure D1.1 *Effect of cation mixtures with biologically-relevant compositions on DNA-membrane bridging. The bar plot shows the mean fluorescence intensity of the Cy3-labelled DNA duplex (36 bp) coating DPPC vesicles (gel phase) in various solutions with added salt (Na^+ with Mg^{2+} or Ca^{2+}). The graphical legend refers to the compositions reported in the representative confocal micrographs, illustrating fluorescence intensity of the detected DNA coating at equatorial sections of the vesicles. Micrographs are presented with post-processing contrast enhancement to enable the visualization of the differences in membrane attachment. For two of the micrographs, surrounded by grey frames, contrast was additionally increased post-processing in order to emphasize the absence of DNA adhesion (sodium only) or only the presence of a faint DNA layer (1 mM Mg^{2+} in the presence of sodium), which would not be detected in the intensity settings used for other samples. Data analysis (presented on the column plot) was done on micrographs without any post-processing, all imaged with the same microscope settings. Scale bars: 10 µm.*

We have then carried out analogous experiments exploring the competition of sodium with calcium, instead of magnesium. Fluctuations in Ca^{2+} concentration bear a particular biological relevance owing to the pivotal role that intercellular calcium waves play in coordinating cellular responses[32]. We have observed that, when present at the same concentrations tested for Mg^{2+}, Ca^{2+} induces a much stronger bridging action, consistently with previous observations of lower sensitivity to the competing effect of monovalent cations[33].

The results presented in Supplementary Fig. D1.1 highlight how the mechanisms for ion-mediated DNA-lipid interactions are sensitive not only to ion concentration, but also to ion identity and the composition of ionic mixtures. For instance, our findings suggest that in certain ion mixtures an increase in calcium content could induce DNA attachment, while a similar change in magnesium concentration will not generate a strong response. It is thus possible to envisage updates in the design of DNA nanostructures specifically aimed at enhancing ion-selectivity, opening up a whole new range of possible targeting mechanisms and responses, e.g. sensitive to ion-mediated signalling and enzymatic action[34]. Additionally, the nanodevices could be made sensitive to deregulated ion transport, which is often a hallmark of a damaged cellular functioning, with altered Ca^{2+} signalling in cancer cells being one particularly illustrative example[35].

Supplementary Discussion 2. Zeta potential measurements

Supplementary Fig. D2.1 shows the results from zeta (ξ) potential measurements on DPPC large unilamellar vesicles (LUVs, Ø (246 ± 118) nm, Supplementary Fig. D2.2) above and below the melting temperature. All the values are collected in Supplementary Table 4.

Despite the zwitterionic nature of the PC head group, we observe a negative ξ-potential (-12.2 ± 0.2 mV) for the LUVs in solution with no added salt. The net surface charge is thought to emerge from the orientation of the PC head group, in which the positively charged choline moiety arranges itself towards the bilayer core, leaving the anionic phosphate more exposed to the solvent[36,37]. The negative surface charge explains the lack of DNA attachment in the absence of cations[38]. After the addition of magnesium ions, the surface charge is screened (ξ-potential = -0.5 ± 0.1 mV), suggesting cation adsorption to the surface, which may in turn lead to DNA adsorption through bridging. Once the system is heated up, causing the vesicles to transition to the liquid phase, the ξ-potential drops back to -9.0 ± 1.0 mV, indicating magnesium desorption. The reversible attachment of DNA can also be observed via ξ-potential measurements: the ξ-potential of DNA-coated vesicles reaches -2.7 ± 0.2 mV, and drops to -7.1 ± 0.7 mV after heating the samples above the transition temperature of the membranes.

Measurements performed on liquid-phase POPC vesicles in the absence of salts, however, result in a value of ξ-potential similar to the one recorded for gel-phase DPPC, suggesting that factors other than surface charge regulate adsorption of cations onto PC bilayers, and thus DNA adhesion.

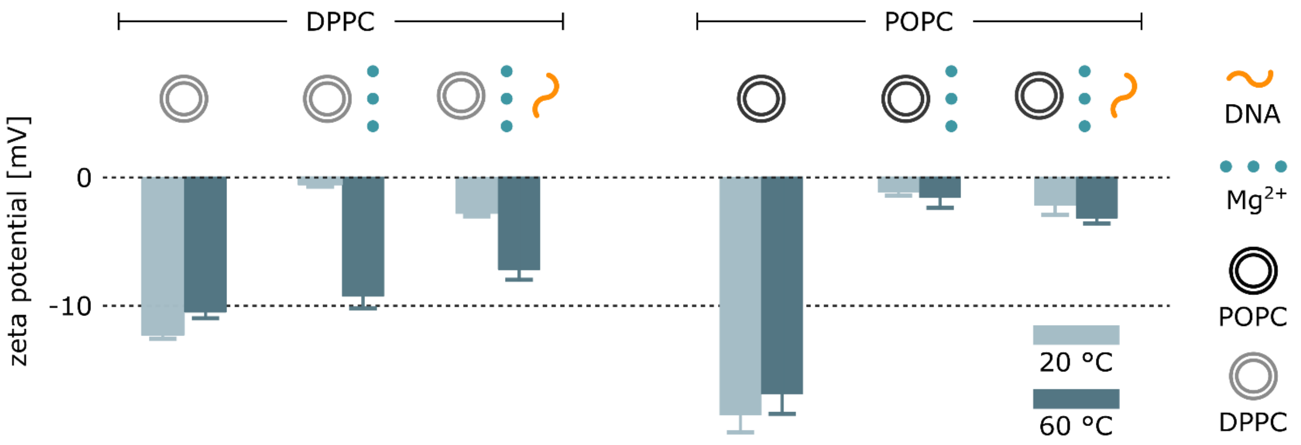

Supplementary Figure D2.1 *Zeta (ξ) potential values collected for either DPPC or POPC LUVs in the solution with no salt, after addition of* Mg^{2+} *(1 mM) and DNA (10 nM). Values were measure below (20°C) and above (60°C) the DPPC phase-transition temperature. Error bars represent standard deviation from three measurements, each consisting of 12 runs. ξ-potential values for DPPC LUVs in the presence (or absence) of* Mg^{2+} *and DNA constructs at different temperatures highlight the electrostatic notions at play in the observed attachment: cations, which screen the strong negative surface charge of the bilayers at room temperature, seem to undergo desorption at high temperatures. On the other hand, the values obtained for POPC LUVs readily indicate that these liquid-phased bilayers also display a strong negative surface charge, screened in the presence of cations. The latter therefore suggests that other factors play a role in the adhesion of DNA constructs to gel-phase vesicles.*

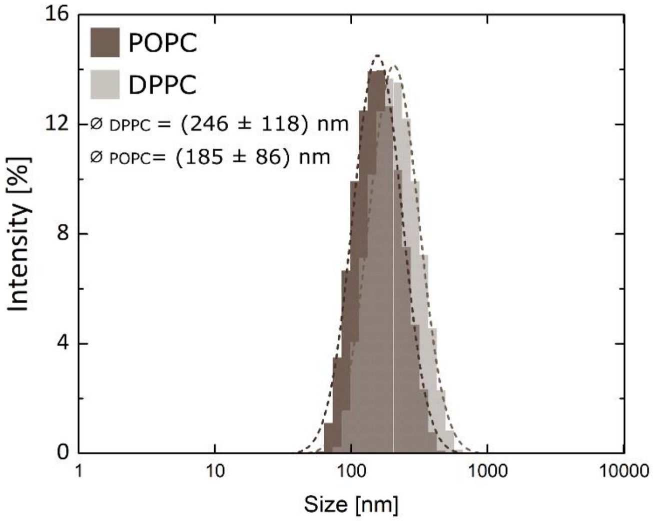

Supplementary Figure D2.2 *Size histogram of LUVs used for zeta-potential measurements, summarised in Supplementary Figure D2.1, collected via dynamic light scattering (DLS). The dashed lines represent lognormal fits, with the peak values (± standard deviation) stated on the graph. Histograms are an average of three measurements, each consisting of 10 runs.*

Supplementary Discussion 3. Effects of biologically-relevant cations on DNA-lipid screening

We have performed additional studies to compare the ability of all four major biologically-relevant cations, monovalent potassium (K^+) and sodium (Na^+), and divalent magnesium (Mg^{2+}) and calcium (Ca^{2+}), to modulate the attractive interaction between ss1C constructs and POPC bilayers through screening. Comparing the two divalent ions (Supplementary Fig. D3.1a), we have established that Ca^{2+} has higher screening efficiency than Mg^{2+}, in agreement with the previously observed higher affinity of calcium for DNA[33]. Similarly, sodium has higher screening efficiency than potassium, again consistently with the greater affinity of Na^+ for nucleic acids[39–41]. The difference is possibly also influenced by the greater tendency of K^+ to form clusters with Cl^-, hence reducing the number of potassium cations available for DNA binding[39]. Interestingly, comparing the behavior of cation species of the same valency shows that their different affinities agree with their order in the Hofmeister series (by ion-hydration: $Na^+ > K^+$, $Ca^{2+} > Mg^{2+}$)[42], which classifies ions with respect to their effects on proteins. Such agreement can hint on the involved molecular phenomena, and also suggests another point in which the similarity between DNA and proteins can be exploited for mimicking membrane structures.

Importantly, the concentrations of the two monovalent cations differ between biological environments, with potassium dominating in intra-cellular and sodium in inter-cellular fluids (Supplementary Fig. D3.1b). Therefore, the observed differences in screening efficiency, leading to different levels of DNA-lipid interactions, can be potentially exploited for *in vivo* nanotechnological applications, and bear potential relevance for biological phenomena.

The histograms in Supplementary Fig. D3.1c present the fluorescence intensity measured from a cholesterol-modified DNA construct (ss1C) for concentrations of monovalent cations approximately representing those encountered in key cellular compartments (5, 10 and 100 mM). In this respect, mitochondria and nuclei are of particulate interest, as the former exhibit lower[43,44], and the latter higher[31,45] cation concentration compared to the cytoplasm. These differences could be directly exploited in further studies to construct DNA probes that target specific organelle membranes. Note, however, that experiments shown here are performed on model, single-component bilayers, so further investigations would be required to address the potential effects arising for the complex chemistry of biological membranes.

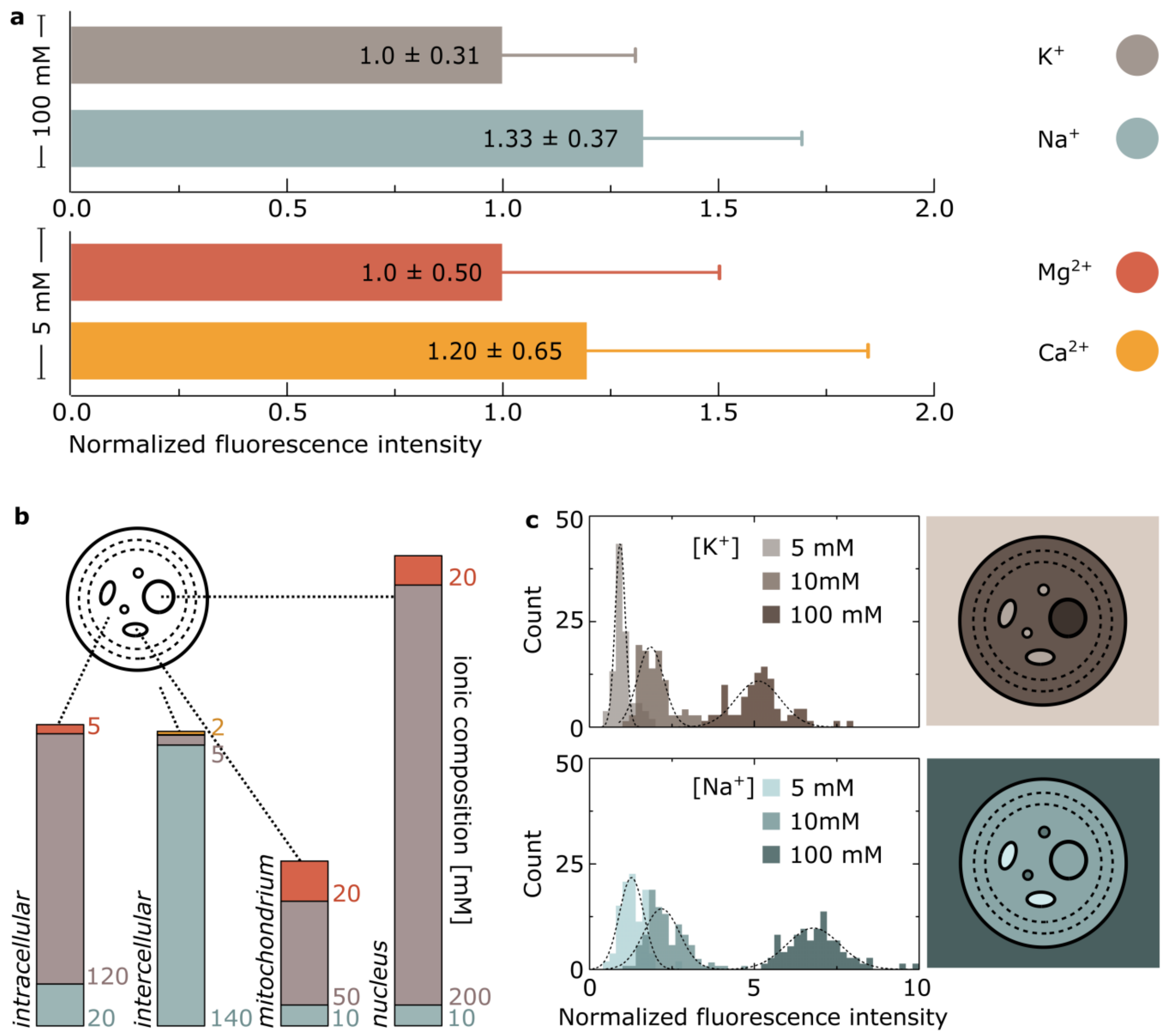

Supplementary Figure D3.1 *Screening effect of biologically relevant ions on DNA-membrane attachment. (a) Normalized fluorescence of cholesterol-modified DNA (0.1 µM, ss1C) coating POPC vesicles (liquid phase) in the presence of 100 mM of monovalent ions: either sodium or potassium (normalized to the peak value of the distribution in K^{+}), or in 5 mM of divalent ions: magnesium and calcium (normalized to the peak value of the distribution in Mg^{2+}). (b) Illustration of ionic composition of environments in major compartments of the cell: intercellular, cytoplasm, in a mitochondrion and in a nucleus[31]. Values below 1 mM have not been stated on the plot for clarity. (c) Histograms of fluorescence intensities from membrane-attached DNA as measured in GUVs incubated with monovalent ion concentrations (5, 10, and 100 mM) approximately as found in cellular fluids (cytoplasm, mitochondria, nuclei, and intercellular). Dashed lines represent Gaussian fits to each histogram. All histograms were normalized to the peak value of the distribution of experiments containing 5 mM K^{+} (the lowest here). Sketches of cells are color-coded (following the plot legend) to illustrate the differences in the concentration of respective monovalent ions in cellular compartments.*

Supplementary Discussion 4. Charge screening studied with PAGE

One may argue that the observed dependency of the fluorescent intensity of membrane-adsorbed DNA on cation concentration might be influenced by artefacts associated with the decreased stability of the DNA duplexes at low ionic strength. Indeed, for both the duplex nanostructures (1C and 2C), the cholesterol(s) and the fluorophores are covalently linked to separate single-stranded components (Supplementary Fig. 1), thus if the nanostructures were to disassemble at low ionic strength one could still have non-fluorescent cholesterol-modified DNA localising on the membranes, and the hydrophilic fluorescent strands remaining in solution.

We ruled out this artefact by performing PAGE analysis, showing that at the studied magnesium concentrations the majority of DNA strands are indeed assembled into duplexes (Supplementary Fig. D4.1). Representative intensity profiles recorded from two different gels at 0 and 4 mM Mg^{2+} are shown in Figure 3c, for the case of 2C motifs. In the absence of salt, a small proportion of the constructs did not assemble properly (grey triangle), however most of the strands - 85% - formed the designed 48-bp duplex (turquoise triangle), as indicated by the size reference. Expectedly, single-stranded components were not observed in samples prepared at 4 mM Mg^{2+}. The difference in migration distance recorded in the two different gels, and explored further in Supplementary Fig. D4.2, is ascribed to the effect that shielding the negatively-charged phosphate has on the rate of migration[46] – yet another illustration of the nucleic acid charge screening.

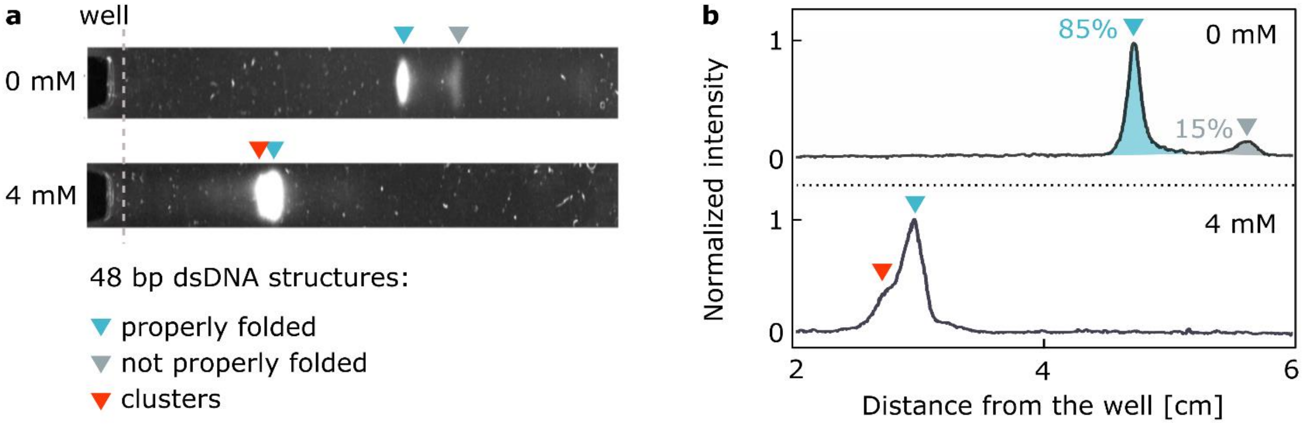

Supplementary Figure D4.1 *(a) PAGE of DNA nanostructures (2C) containing either 0 or 4 mM $MgCl_2$ in both the gel and the buffer. The dsDNA constructs self-assemble correctly, as indicated by the blue arrow, incorrectly (grey arrow) and cluster (red arrow). Sodium ions ($\approx$ 10 mM) were present in all samples. (b) Inverted intensity profiles of the gel lanes shown in (a).*

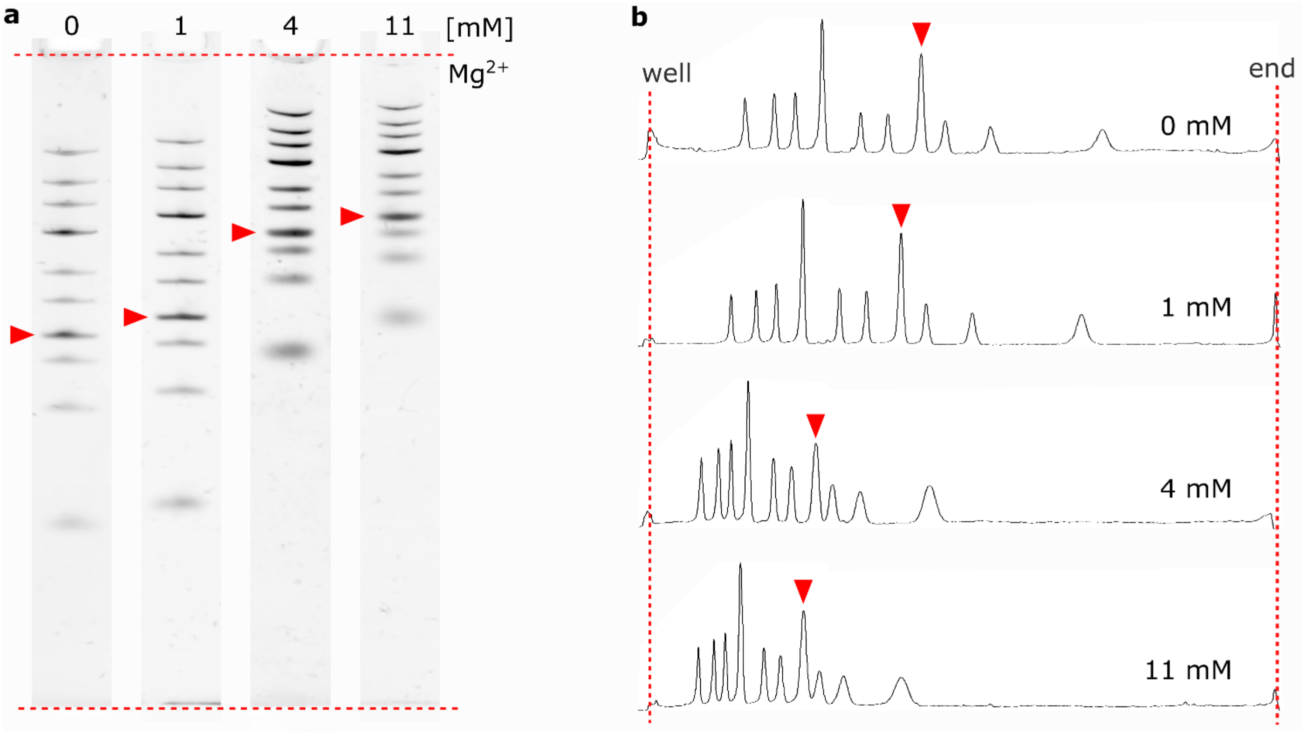

Supplementary Figure D4.2 *Charge screening modulated DNA mobility in PAGE (a) DNA reference ladder (GeneRuler Low Range) run in gels with 0, 1, 4, 11 mM Mg^{2+} using the same settings. Red dashed lines represent the edges of the gel (well and the end). Red markers indicate the position of the 100 bp band of the ladders. (b) Intensity traces of the respective ladders from (a). Symbols as in (a). The differences in mobility are visible from the shift in position of the bands, and result from positive ions in the solution screening the negatively-charged phosphates on DNA molecules and thus affecting its response to the applied electric field.*

S11 Figures

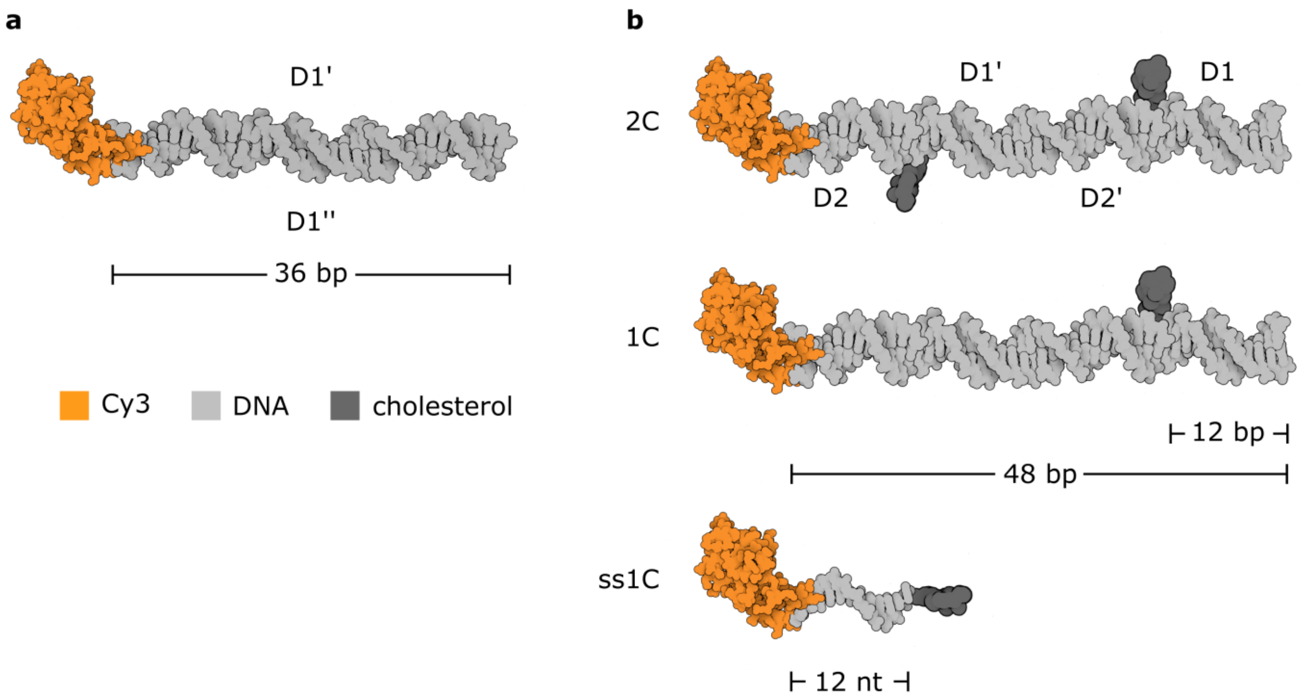

Supplementary Figure 1. Sketches of DNA constructs used it this work. (a) The 36-bp long duplex, with D1’ strand modified with Cy3 molecule, used for the study of phase- and cation-dependent membrane/DNA affinity (Fig. 1). (b) The three cholesterol-modified nanostructures used to study the effect of ionic screening on hydrophobicity-induced membrane insertion in liquid membranes (Fig. 3 and 4): 48-bp long duplexes, consisting of four strands, with two (2C) or one (1C) cholesterol moiety, respectively, alongside a ssDNA strand of 12 nucleotides, modified on both ends with either Cy3 or cholesterol.

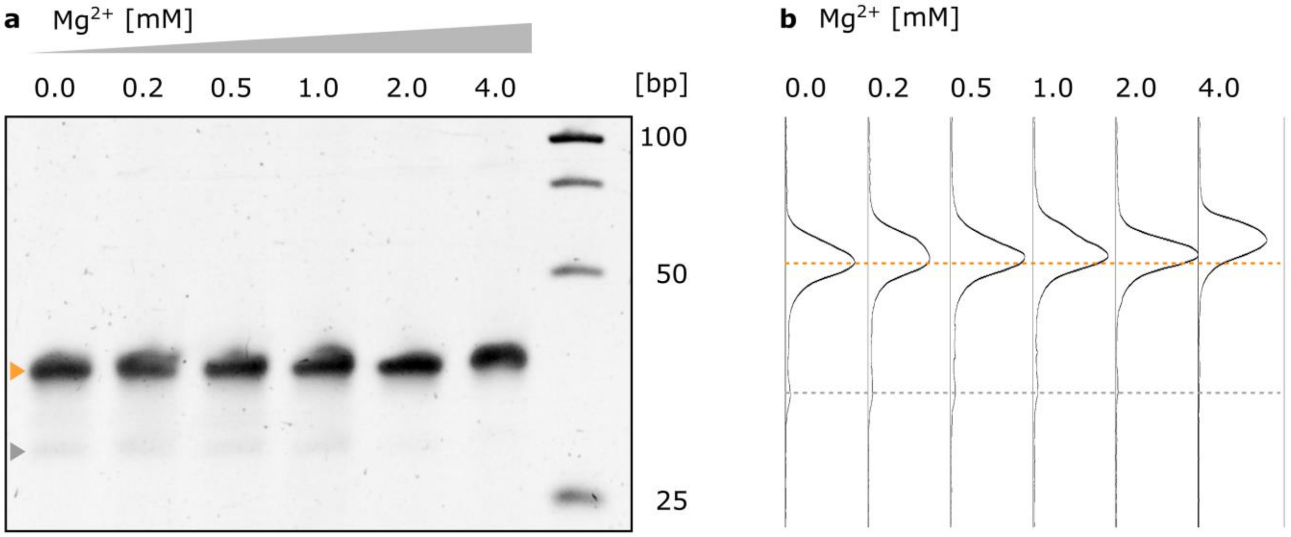

Supplementary Figure 2 PAGE of the 36 bp-long duplex (Supplementary Fig. 1a) used to probe the phase- and cation-dependent DNA/membrane attachment in various Mg^{2+} concentrations. The (intensity-inverted) image of the gel, highlighting the folded duplex (orange) and residual single-stranded components (grey). (b) The intensity profiles of the lanes seen in (a). Dashed lines correspond to the position of markers on the gel. Sodium ions (˜10 mM) were present in each sample. The gel and the buffer did not contain divalent cations.

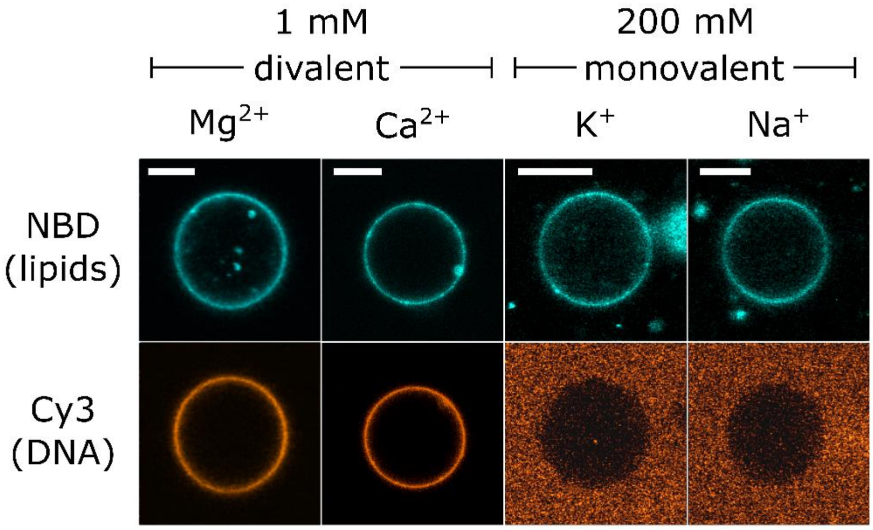

Supplementary Figure 3. Representative micrographs showing the attachment of the unmodified DNA duplex (Supplementary Fig. 1a) to GUVs in solutions containing different cations. Vesicles were prepared with DPPC and 0.5% of NBD-PC. Images, acquired at room temperature with membranes in gel phase, showcase the selective attachment that DNA duplexes display for gel-phases only in the presence of divalent cations. Scale bar: 10 µm.

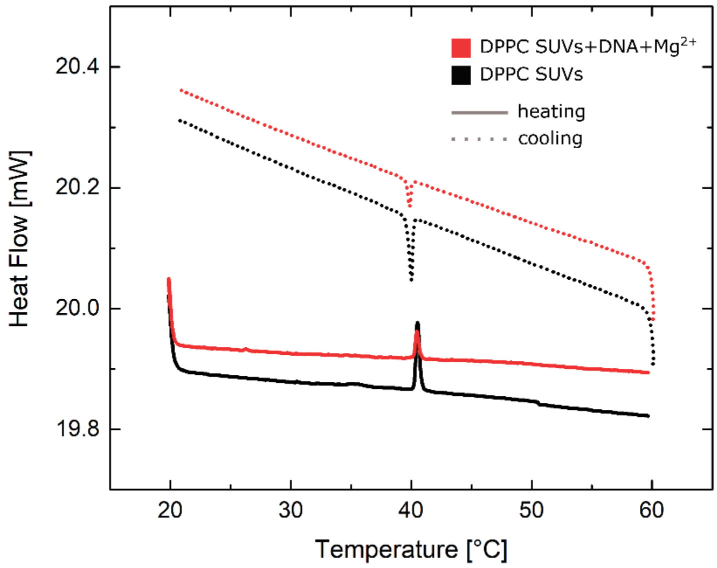

Supplementary Figure 4. Differential scanning calorimetry (DSC) measurements confirming DPPC phase transition temperature (41 °C [47]). Experiments were performed with LUVs in solution without added salt, as well as in the presence of Mg^{2+} and DNA, as for the experiments on DNA-membrane attachment in Fig. 1.

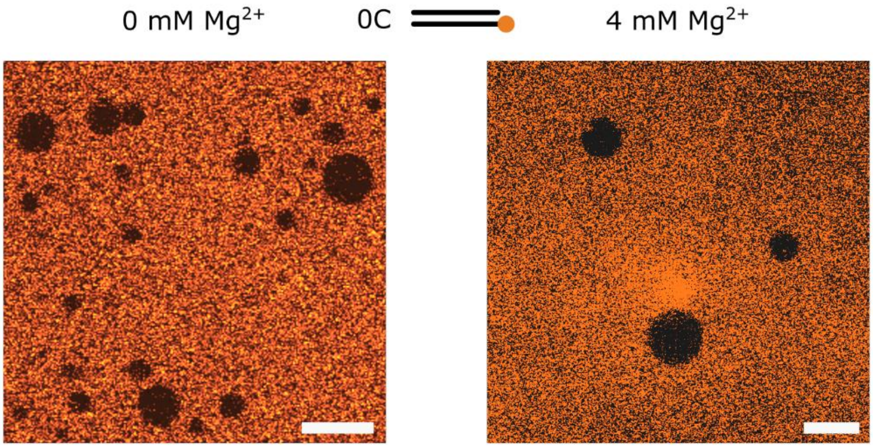

Supplementary Figure 5. Representative confocal micrographs illustrating the lack of attachment of unmodified DNA duplexes (Supplementary Fig. 1a) to POPC vesicles, regardless of the presence of cations. POPC vesicles, imaged at room temperature, display a liquid disordered phase, further highlighting the role of phase in the cation-mediated bridging between the bilayer and the DNA molecules. Scale bars represent 20 µm.

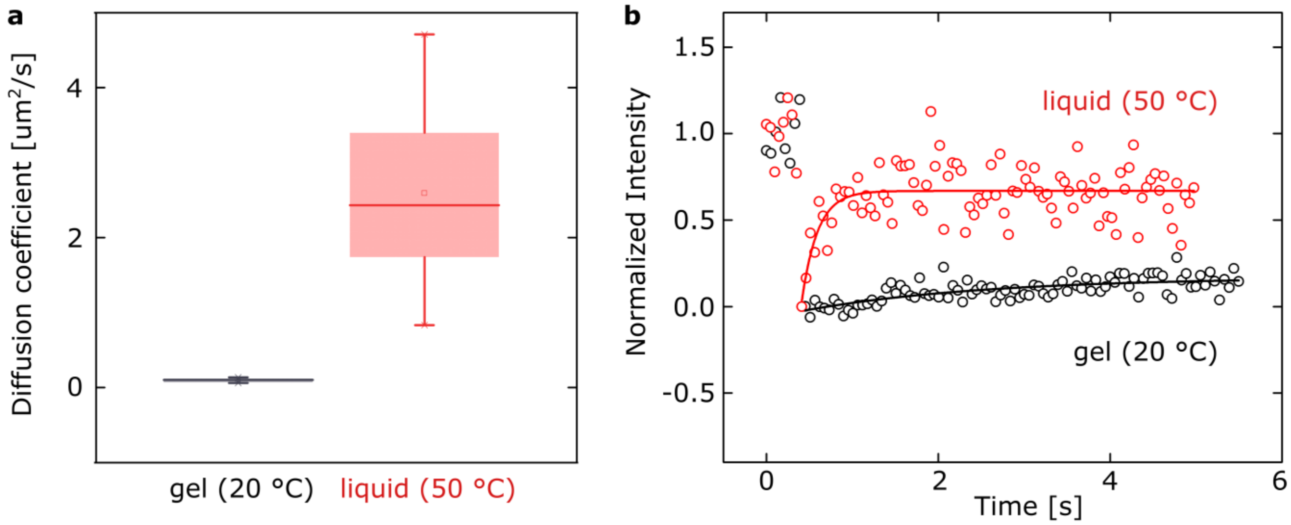

Supplementary Figure 6. FRAP measurements confirmed the phase of DPPC vesicles. (a) Box plots showing the diffusion coefficient (D) values for fluorescently tagged NBD-PC lipids in DPPC gel (D = 0.10 ± 0.03 um^2/s) and liquid (D = 2.59 ± 1.24 um^2/s) phase membranes. (b) Representative traces of fluorescence after bleaching, illustrating negligible diffusion of lipids in their gel phase.

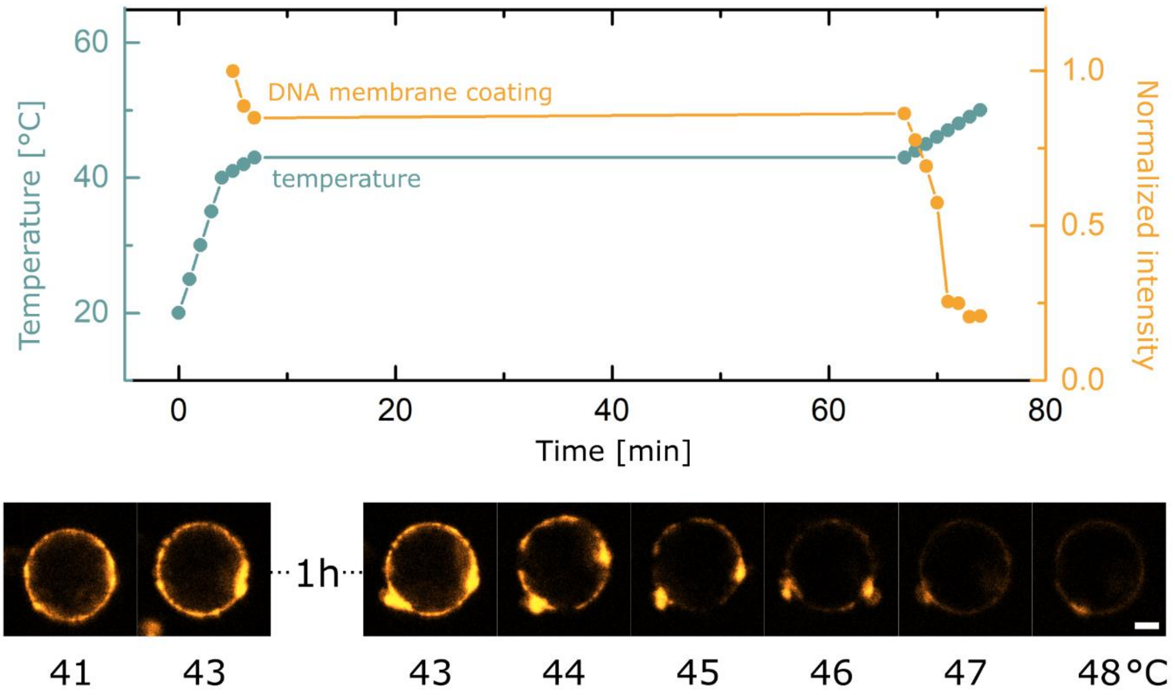

Supplementary Figure 7. Plot illustrating the time evolution of the fluorescence intensity emitted by Cy3-labelled 36 bp duplex DNA coating a DPPC GUV following temperature changes. While a degree of DNA detachment occurs rapidly after the temperature is increased above T_m = 41 °C, incubating the sample at constant temperature (43 °C) for 1 h leads to no changes in the coating, confirming the absence of a slow detachment kinetics. Upon further temperature increase, the detachment proceeded. Representative confocal micrographs confirm the absence of time-dependent detachment of DNA duplexes (Supplementary Fig. 1a) from DPPC GUVs if incubated just above T_m. Scale bar represents 5 µm.

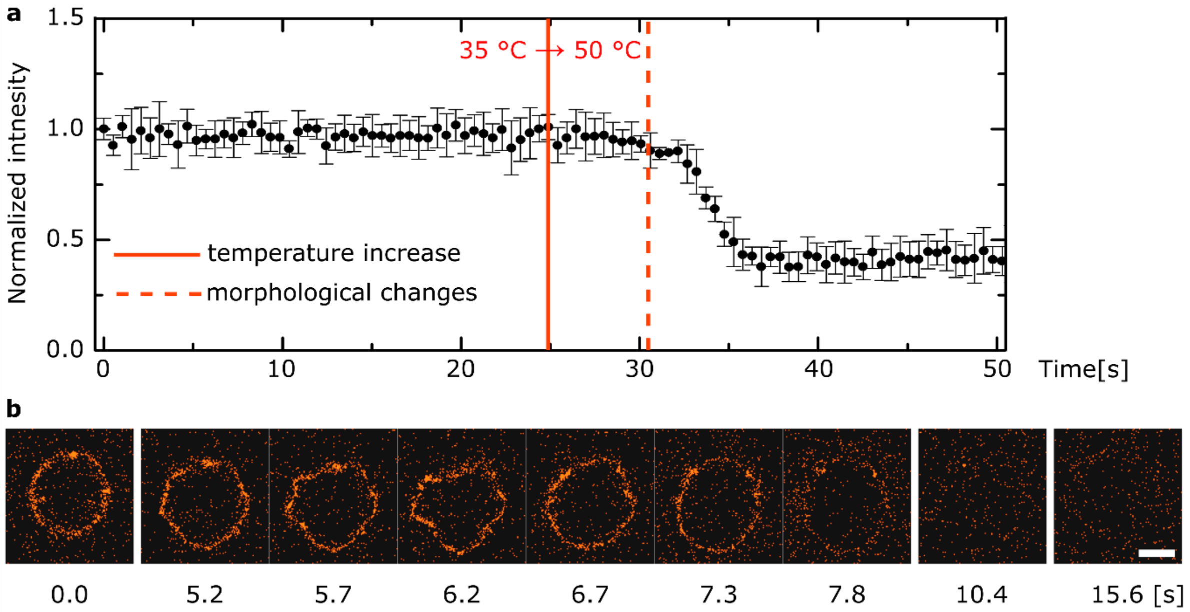

Supplementary Figure 8. Fluorescence signal of a non-cholesterolized, Cy-3 labelled DNA duplex (36 bp) coating of a DPPC vesicle changing with time, upon fast temperature increase from below to above the DPPC phase transition temperature T_m = 41 °C (35 °C to 50 °C). (a) Plot showing the time-dependent fluorescent signal, highlighting desorption after the lipid phase transition is induced by the quick temperature change. Expectedly, and due to heat propagation in our microscopy cell, a slight delay of ~5 s is observed between the triggered temperature change (solid vertical line) and the lipid-membrane phase transition (dashed vertical line). The latter is identified from abrupt morphological changes in the GUVs, as illustrated in panel b. Error bars represent standard deviation of the signal recorded for two vesicles. (b) Representative micrographs illustrating changes summarized by the plot in (a). Around 5 s after the temperature was increased morphology of the vesicle changed, indicating phase transition. Scale bar: 10 µm.

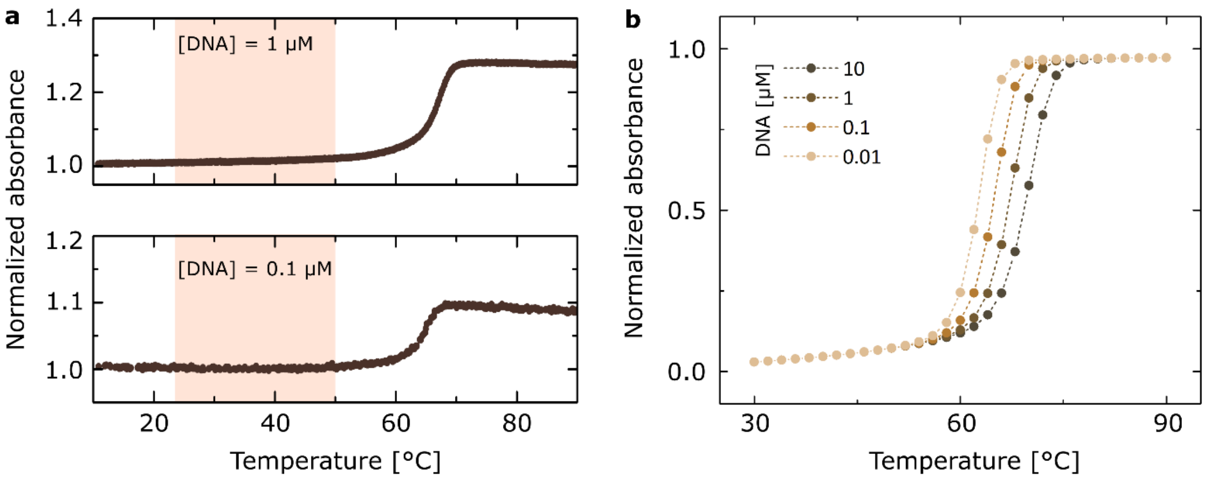

Supplementary Figure 9. The DNA duplex probe (36 bp, Cy3-labeled) is stable in the temperature range relevant for experiments in Fig. 1. (a) Melting profiles (absorbance at 260 nm) collected for the 36 bp duplex used in the temperature-controlled experiments. Measurements of the sample containing 1 and 0.1 µM DNA were conducted in the presence of 1 mM $MgCl_2$, and in both cases the melting transition is found above the temperature range relevant for the experiments summarized in Fig. 1c: [20,50] °C (highlighted in orange). The data were normalized to the initial value of each set. (b) Melting profiles obtained for the same structure via NUPACK[1] analysis, showing that with increasing DNA concentration the melting temperature also increases. Given the adhesive effect driven by Mg^{2+}-mediated bridging, we expect the concentration of DNA to be higher at the membrane interface than in the solution (0.1 µM), which further supports ruling out temperature instability as a possible source of artefacts impacting our results.

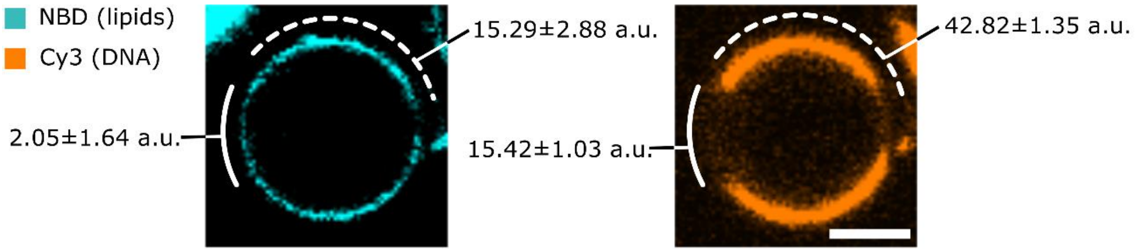

Supplementary Figure 10. Co-localization of DNA nanostructures and NBD-PC in de-mixed 15% cholesterol/DPPC vesicles. Representative micrographs, alongside average intensity values of the two phases (liquid ordered and gel) stated in arbitrary units, illustrate the lateral partitioning of NBD-PC lipids. The co-localization of the fluorescent signals from the DNA and NBD suggests that they both tag the gel-phased domains of the membrane. Scale bar represents 5 µm.

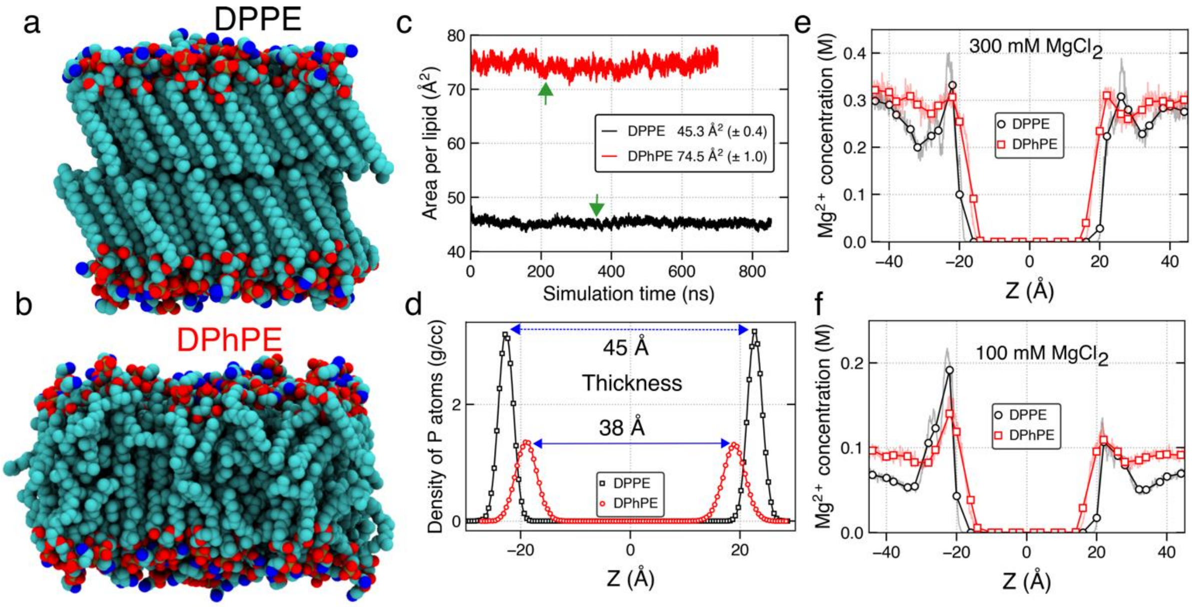

Supplementary Figure 11. Free-equilibration simulation of fluid and gel-phase PE membranes. Representative conformation of the (a) gel-phase (DPPE) (b) fluid-phase (DPhPE) lipid bilayer membranes containing 64 lipid molecules in each leaflet. The non-hydrogen atoms of the lipid bilayer membrane are shown as blue (N), tan (P), red (O), and cyan (C) spheres. (c) The area per head group of the DPhPE and DPPE membranes simulated first in 300 mM (up to the green arrow) and later in 100 mM solution of $MgCl_2$. (d) Local density of the P atoms of the lipid headgroups along the bilayer normal averaged over the last 0.5 µs of the equilibrium MD simulations. Local concentration of Mg^{2+} ions along the lipid bilayer normal averaged over the respective MD trajectory (excluding the first 50 ns) for the simulations performed at (e) 300 mM and (f) 100 mM concentration of $MgCl_2$. Symbols show local concentration data averaged in 3 Å bins, faded lines show 0.1 Å bin data.

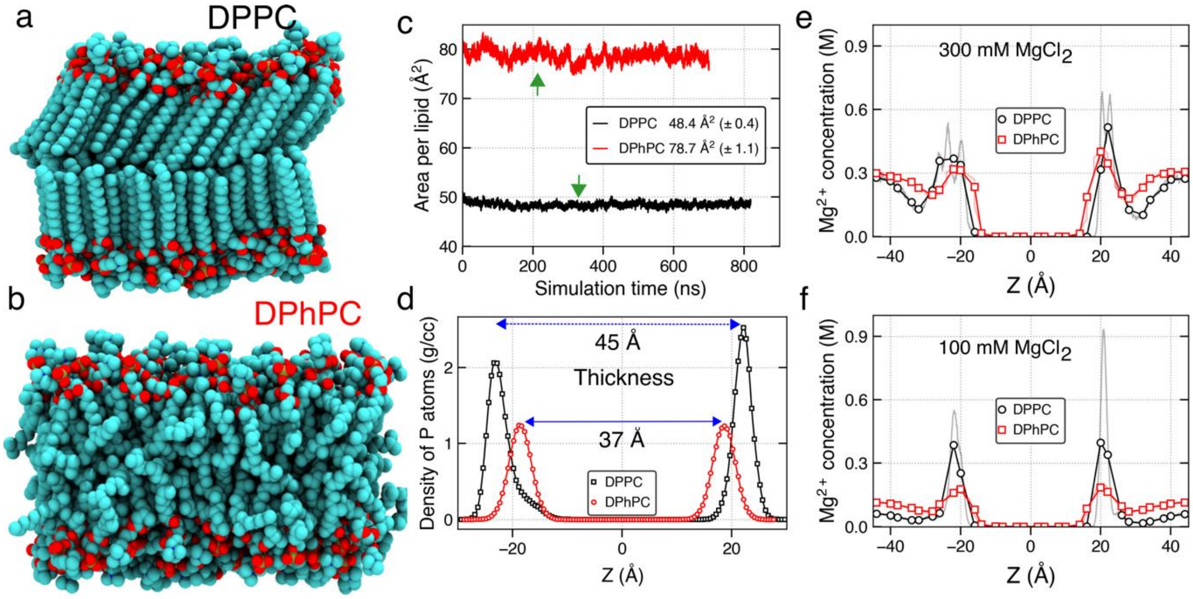

Supplementary Figure 12. Free-equilibration simulation of fluid and gel-phase PC membranes. Representative conformation of the (a) gel-phase (DPPC) (b) fluid-phase (DPhPC) lipid bilayer membranes containing 64 lipid molecules in each leaflet. The non-hydrogen atoms of the lipid bilayer membrane are shown as blue (N), tan (P), red (O), and cyan (C) spheres. (c) The area per head group of the DPhPC and DPPC membranes simulated first in 300 mM (up to the green arrow) and later in 100 mM solution of $MgCl_2$. (d) Local density of the P atoms of the lipid headgroups along the bilayer normal averaged over the last 0.5 µs of the equilibrium MD simulations. Local concentration of Mg^{2+} ions along the lipid bilayer normal averaged over the respective MD trajectory (excluding the first 50 ns) for the simulations performed at (e) 300 mM and (f) 100 mM concentration of $MgCl_2$. Symbols show local concentration data averaged in 3 Å bins, faded lines show 0.1 Å bin data.

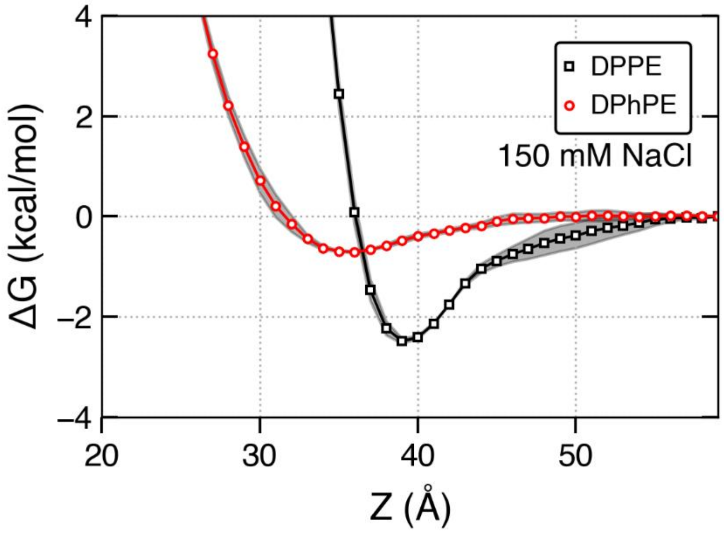

Supplementary Figure 13. Free energy of the 21 base pair DNA fragment versus its distance to the membrane midplane for 150 mM NaCl solution.

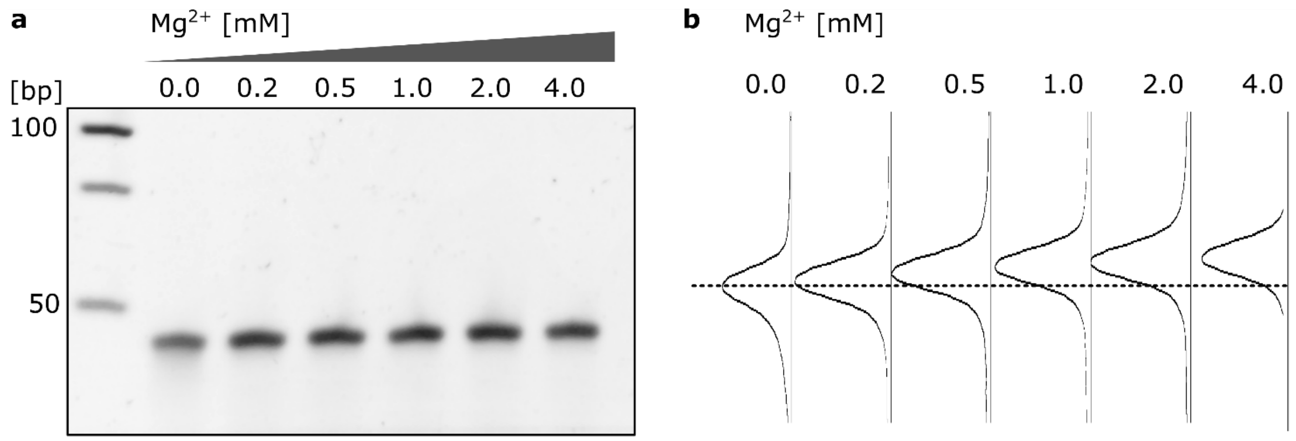

Supplementary Figure 14. PAGE of unmodified (2C) DNA construct folded in different concentration of magnesium. (a) Intensity-inverted image of the gel, indicating presence of folded structure in each sample. (b) The intensity profiles of the lanes of the gel. The dashed line represents the position of structures folded in the absence of magnesium. Sodium ions ($\approx$ 10 mM) were present in all samples.

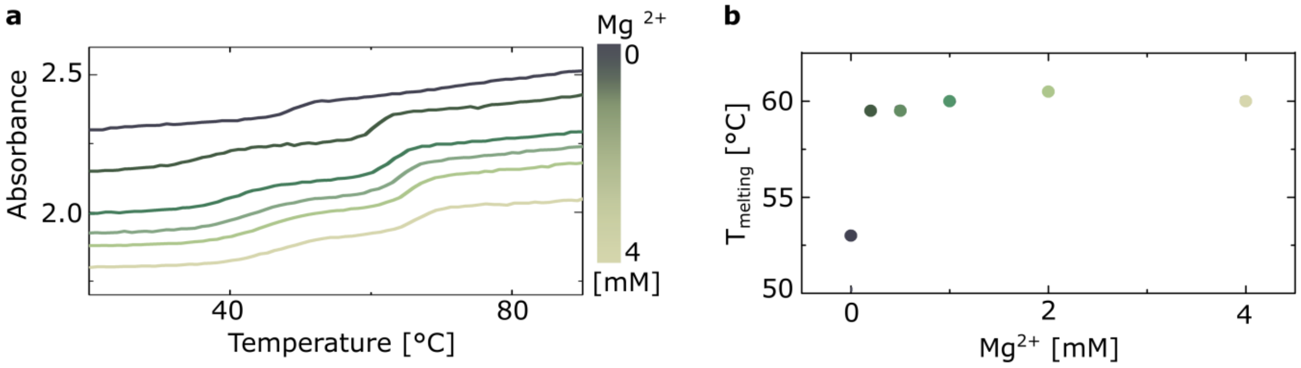

Supplementary Figure 15. Mg^{2+}-dependency of melting temperatures of unmodified (1C/2C without cholesterol) DNA construct. (a) Collected UV-vis absorbance at 260 nm. Two-steps profile results from the structure's design, with two short terminal strands disconnected at lower temperatures than the two longer ones. (b) Melting temperatures plotted against concentration of Mg^{2+} in the sample. Experiments were performed in 1x TE buffer, with the respective concentration of ions. Sodium ions (~10 mM) were present in all samples.

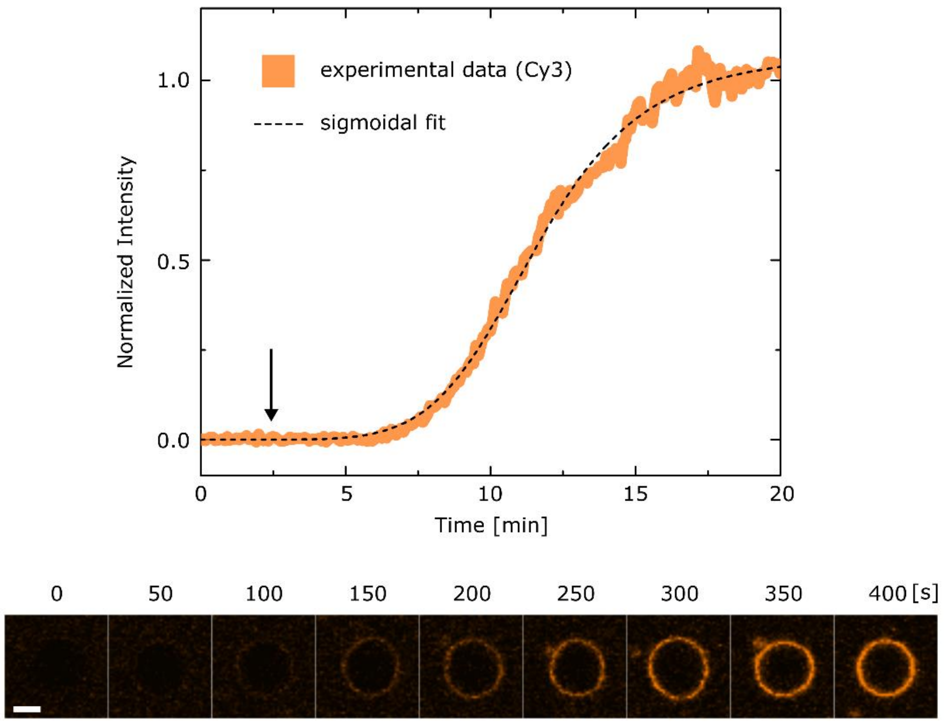

Supplementary Figure 16. Calcium-triggered DNA membrane attachment of nanostructure 2C. The plot shows changes in intensity of the fluorescent DNA ring coating the vesicle. The addition of calcium (final [Ca^{2+}] = 4 mM) is marked with an arrow. Scale bar: 5 µm.

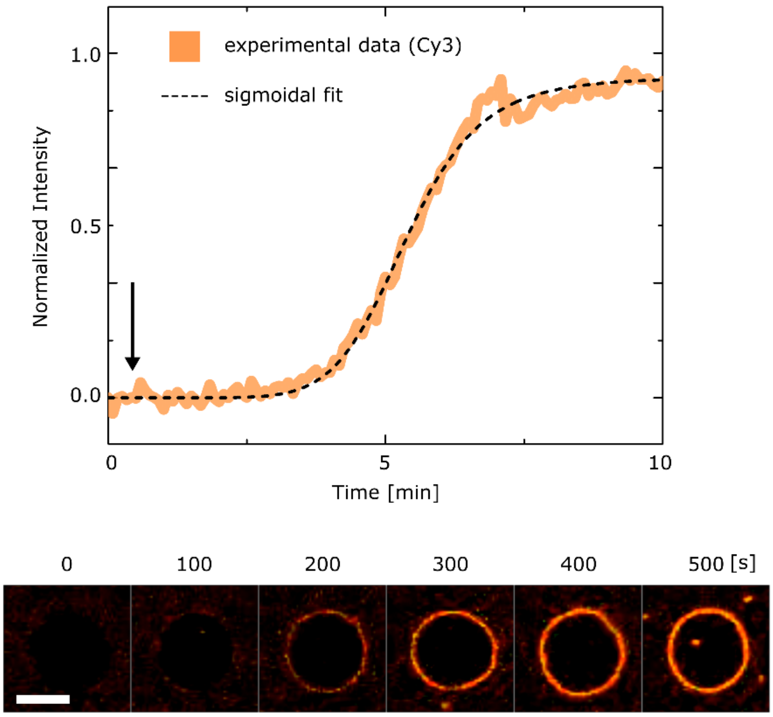

Supplementary Figure 17. Potassium-triggered DNA membrane attachment of nanostructure 2C. The plot shows changes in intensity of the fluorescent DNA ring coating the vesicle. The addition of potassium (final [K^+] = 500 mM) is marked with an arrow. Scale bar:10 µm.

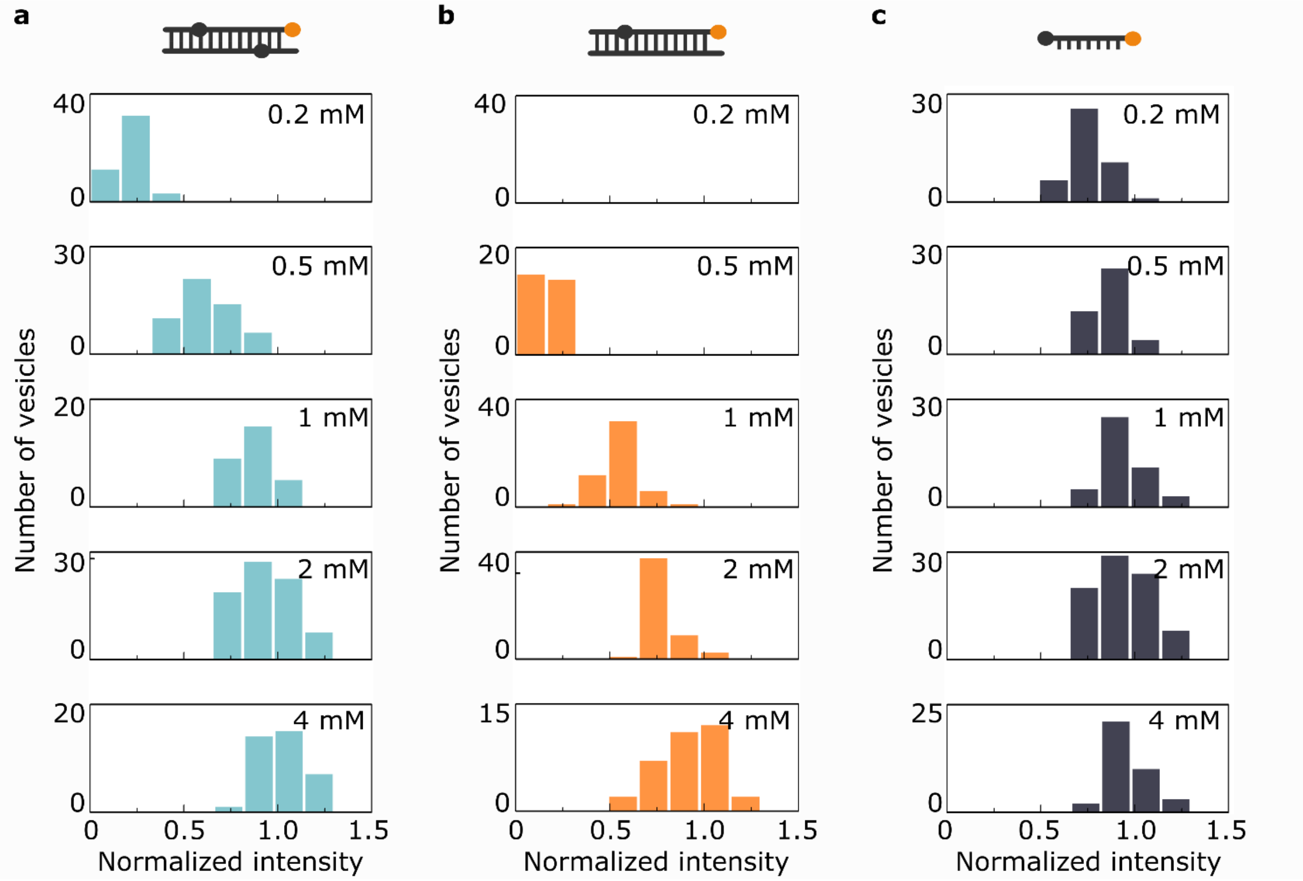

Supplementary Figure 18. Histograms of the fluorescence intensity of DNA-coated vesicles in different magnesium concentrations. (a) 48-bp long structure (2C) modified with 2 cholesterols (represented as black circles). (b) 48-bp long structure (1C) modified with 1 cholesterol. (c) 12-bp long ssDNA modified with 1 cholesterol (ss1C). The mean intensities for each of these structures and conditions are collated in Fig. 3b in the main text. The three DNA constructs bear Cy3 as fluorophore, depicted as a circle in orange.

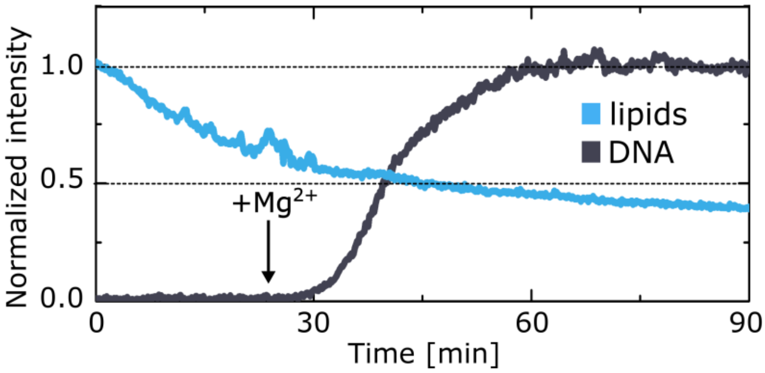

Supplementary Figure 19. Lipid-flipping activity is only induced by membrane-spanning DNA nanostructures. Representative fluorescence trace of NBD-labelled lipids (blue) upon addition of dithionite, alongside the trace of Cy3-labelled (1C) DNA construct (black), coating the vesicle after addition of magnesium (arrow). Note that, unlike its analogue 2C, the 1C nanostructure does not insert into membranes, and thus does not create a toroidal pore. Therefore, after DNA attachment, significant bleaching of NBD is not observed. This assay serves as a control for the Mg^{2+}-triggered flipping activity experiment (Figure 4d-f).

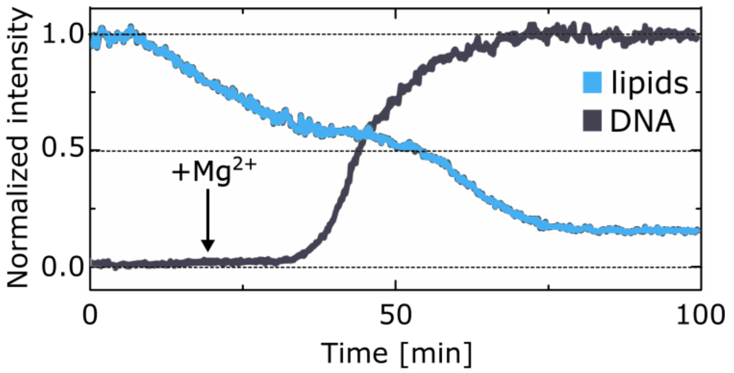

Supplementary Figure 20. An additional example of vesicle's fluorescence indicative of flipping switching with Mg^{2+}. Fluorescence intensity trace of NBD-labelled lipids (blue) upon addition of dithionite, alongside the trace of Cy3-labelled (2C) DNA construct (black), coating the vesicle after addition of magnesium (arrow).

S12 Tables

Table 1. Sequences of DNA strands used in this work.

Strand	*Sequence (5' > 3')*	*Length [bp]*	*Modification*
D1	AGTAGTATCCAT	12	3' TEG Cholesterol, 5' Cy3
D1'	CATCGTAGCTAAAAAAGTCATACATAGATTAGAGAG	36	5' Cy3
D1''	CTCTCTAATCTATGTATGACTTTTTTAGCTACGATG	36	
D2	CTCTCTAATCTA	12	3' TEG Cholesterol
D2'	TGTATGACTTTTTTAGCTACGATGATGGATACTACT	36	

Table 2. Parameters obtained by fitting a Hill equation to the data for DNA-GUV affinity as a function of magnesium concentration, shown in Fig. 3b.

Strand	*nt*	*nt:chol*	*n*	*K [mM]*	*K_d [mM]*
ss1C	12	12	0.72 ± 0.20	0.048 ± 0.014	0.112 ± 0.081
2C	96	48	1.99 ± 0.11	0.389 ± 0.012	0.153 ± 0.002
1C	96	96	2.26 ± 0.65	0.920 ± 0.142	0.828 ± 0.044

Table 3. Average fluorescence intensity from the DNA coating of vesicles incubated in varying magnesium concentrations for the structures tested in Fig. 3 and shown in Supplementary Fig. 1. The values were normalised to the peak value for 2C intensities in 4 mM magnesium.

[Mg^{2+}] [mM]	*ss1C*	*2C*	*1C*
0.2	0.76 ± 0.08	0.21 ± 0.08	0 (no attachment)
0.5	0.85 ± 0.14	0.62 ± 0.14	0.17 ± 0.07
1	0.94 ± 0.08	0.87 ± 0.08	0.60 ± 0.11
2	0.96 ± 0.15	0.94 ± 0.15	0.76 ± 0.09
4	0.98 ± 0.18	1.00 ± 0.18	1.00 ± 0.10

Table 4. Zeta (ξ) potential values presented in the Supplementary Fig. D2.1. Standard deviation from three measurements.

Lipid species	*Conditions*	*Zeta (ξ) potential [mV]*		*Standard deviation [mV]*	
		20 °C	*60 °C*	*20 °C*	*60 °C*
DPPC	no added salt	-12.20	-10.44	0.22	0.49
	1 mM Mg^{2+}	-0.54	-8.99	0.11	0.99
	1 mM Mg^{2+} + 10 nM DNA	-2.73	-7.07	0.16	0.73
POPC	no added salt	-18.07	-16.5	1.45	1.64
	1 mM Mg^{2+}	-1.21	-1.63	0.38	0.88
	1 mM Mg^{2+} + 10 nM DNA	-2.20	-3.18	0.84	0.52

References

1. Zadeh, J. N. *et al.* NUPACK: Analysis and design of nucleic acid systems. *J. Comput. Chem.* **32**, 170–173 (2011).
2. Ohmann, A. *et al.* A synthetic enzyme built from DNA flips 107 lipids per second in biological membranes. *Nat. Commun.* **9**, (2018).
3. Gagné, F. *Biochemical Ecotoxicology: Principles and Methods*. *Biochemical Ecotoxicology: Principles and Methods* (2014). doi:10.1016/C2012-0-07586-2.
4. Harvey, L. *et al. Molecular Cell Biology. 4th edition*. *Journal of the American Society for Mass Spectrometry* (2000). doi:10.1016/j.jasms.2009.08.001.
5. Schindelin, J. *et al.* Fiji: An open-source platform for biological-image analysis. *Nat. Methods* **9**, 676–682 (2012).
6. Marco, I. & Van der Biest, O. Polarization measurements from a rotating disc electrode for characterization of magnesium corrosion. *Corros. Sci.* **102**, 384–393 (2016).
7. Kappel, C. & Eils, R. Fluorescence recovery after photobleaching with the Leica TCS SP2. *Confocal Appl. Lett.* (2004).
8. Lipfert, J., Sim, A. Y. L., Herschlag, D. & Doniach, S. Dissecting electrostatic screening, specific ion binding, and ligand binding in an energetic model for glycine riboswitch folding. *RNA* **16**, 708–719 (2010).
9. Chu, V. B., Bai, Y., Lipfert, J., Herschlag, D. & Doniach, S. A repulsive field: advances in the electrostatics of the ion atmosphere. *Curr. Opin. Chem. Biol.* **12**, 619–625 (2008).
10. Draper, D. E. A guide to ions and RNA structure. (2004) doi:10.1261/rna.5205404.
11. Phillips, J. C. *et al.* Scalable molecular dynamics on CPU and GPU architectures with NAMD. *J. Chem. Phys.* **153**, 44130 (2020).
12. Batcho, P. F., Case, D. A. & Schlick, T. Optimized particle-mesh Ewald/multiple-time step integration for molecular dynamics simulations. *J. Chem. Phys.* **115**, 4003–4018 (2001).
13. Feller, S. E., Zhang, Y., Pastor, R. W. & Brooks, B. R. Constant pressure molecular dynamics simulation: The Langevin piston method. *J. Chem. Phys.* **103**, 4613–4621 (1995).
14. Miyamoto, S. & Kollman, P. A. Settle: An analytical version of the SHAKE and RATTLE algorithm for rigid water models. *J. Comput. Chem.* **13**, 952–962 (1992).
15. Andersen, H. C. Rattle: A 'velocity' version of the shake algorithm for molecular dynamics calculations. *J. Comput. Phys.* **52**, 24–34 (1983).
16. Hart, K. *et al.* Optimization of the CHARMM additive force field for DNA: Improved treatment of the BI/BII conformational equilibrium. *J. Chem. Theory Comput.* **8**, 348–362 (2012).
17. Klauda, J. B. *et al.* Update of the CHARMM All-Atom Additive Force Field for Lipids: Validation on Six Lipid Types. *J. Phys. Chem. B* **114**, 7830–7843 (2010).
18. Beglov, D. & Roux, B. Finite representation of an infinite bulk system: Solvent boundary potential for computer simulations. *J. Chem. Phys.* **100**, 9050–9063 (1994).
19. Yoo, J. & Aksimentiev, A. Improved parametrization of Li +, Na +, K +, and Mg 2+ ions for all-atom molecular dynamics simulations of nucleic acid systems. *J. Phys. Chem. Lett.* **3**, 45–50 (2012).
20. Yoo, J. & Aksimentiev, A. Improved Parameterization of Amine-Carboxylate and Amine-Phosphate Interactions for Molecular Dynamics Simulations Using the CHARMM and AMBER Force Fields. *J. Chem. Theory Comput.* **12**, 430–443 (2016).
21. Humphrey, W., Dalke, A. & Schulten, K. VMD: Visual molecular dynamics. *J. Mol. Graph.* (1996) doi:10.1016/0263-7855(96)00018-5.
22. Case, D. A. *et al.* AMBER14. *AMBER 14* (2014).
23. Jo, S., Lim, J. B., Klauda, J. B. & Im, W. CHARMM-GUI membrane builder for mixed bilayers and its

application to yeast membranes. *Biophys. J.* **97**, 50–58 (2009).
24. Jorgensen, W. L., Chandrasekhar, J., Madura, J. D., Impey, R. W. & Klein, M. L. Comparison of simple potential functions for simulating liquid water. *J. Chem. Phys.* **79**, 926–935 (1983).
25. Sugita, Y., Kitao, A. & Okamoto, Y. Multidimensional replica-exchange method for free-energy calculations. *J. Chem. Phys.* **113**, 6042–6051 (2000).
26. Fiorin, G., Klein, M. L. & Hénin, J. Using collective variables to drive molecular dynamics simulations. *Mol. Phys.* **111**, 3345–3362 (2013).
27. Kumar, S., Rosenberg, J. M., Bouzida, D., Swendsen, R. H. & Kollman, P. A. THE weighted histogram analysis method for free-energy calculations on biomolecules. I. The method. *J. Comput. Chem.* **13**, 1011–1021 (1992).
28. Macke, T. J. & Case, D. A. Modeling Unusual Nucleic Acid Structures. *ACS Symp. Ser.* **682**, 379–393 (1998).
29. Carr, R., Comer, J., Ginsberg, M. D. & Aksimentiev, A. Microscopic perspective on the adsorption isotherm of a heterogeneous surface. *Journal of Physical Chemistry Letters* vol. 2 1804–1807 (2011).
30. Higgins, M. J., Tom, L. A. & Sobeck, D. C. Case Study I: Application of the Divalent Cation Bridging Theory to Improve Biofloc Properties and Industrial Activated Sludge System Performance-direct Addition Of Divalent Cations. *Water Environ. Res.* **76**, (2004).
31. Bischof, H. *et al.* Live-Cell Imaging of Physiologically Relevant Metal Ions Using Genetically Encoded FRET-Based Probes. *Cells* **8**, 492 (2019).
32. Leybaert, L. & Sanderson, M. J. Intercellular Ca2+ waves: Mechanisms and function. *Physiological Reviews* vol. 92 1359–1392 (2012).
33. Korolev, N., Lyubartsev, A. P., Rupprecht, A. & Nordenskiöld, L. Competitive binding of Mg2+, Ca2+, Na+, and K+ ions to DNA in oriented DNA fibers: Experimental and Monte Carlo simulation results. *Biophys. J.* **77**, 2736–2749 (1999).
34. Murphy, E. Mysteries of magnesium homeostasis. *Circulation Research* vol. 86 245–248 (2000).
35. Stewart, T. A., Yapa, K. T. D. S. & Monteith, G. R. Altered calcium signaling in cancer cells. *Biochimica et Biophysica Acta - Biomembranes* vol. 1848 2502–2511 (2015).
36. Morini, M. A. *et al.* Influence of temperature, anions and size distribution on the zeta potential of DMPC, DPPC and DMPE lipid vesicles. *Colloids Surfaces B Biointerfaces* **131**, 54–58 (2015).
37. Seelig, J. Deuterium magnetic resonance: Theory and application to lipid membranes. *Q. Rev. Biophys.* **10**, 353–418 (1977).
38. Antipina, A. Y. & Gurtovenko, A. A. Molecular-level insight into the interactions of DNA with phospholipid bilayers: Barriers and triggers. *RSC Adv.* **6**, 36425–36432 (2016).
39. Savelyev, A. & Papoian, G. A. Electrostatic, steric, and hydration interactions favor Na+ condensation around DNA compared with K+. *J. Am. Chem. Soc.* **128**, 14506–14518 (2006).
40. Allahverdi, A., Chen, Q., Korolev, N. & Nordenskiöld, L. Chromatin compaction under mixed salt conditions: Opposite effects of sodium and potassium ions on nucleosome array folding. *Sci. Rep.* **5**, 1–7 (2015).
41. Zinchenko, A. A. & Yoshikawa, K. Na+ shows a markedly higher potential than K+ in DNA compaction in a crowded environment. *Biophys. J.* **88**, 4118–4123 (2005).
42. Metrick, M. A. *et al.* Million-fold sensitivity enhancement in proteopathic seed amplification assays for biospecimens by Hofmeister ion comparisons. *Proc. Natl. Acad. Sci. U. S. A.* **116**, 23029–23039 (2019).
43. Zoeteweij, J. P., Van De Water, B., De Bont, H. J. G. M. & Nagelkerke, J. F. Mitochondrial K+ as modulator of Ca2+-dependent cytotoxicity in hepatocytes. Novel application of the K+-sensitive dye PBFI (K+-binding benzofuran isophthalate) to assess free mitochondrial K+ concentrations. *Biochem. J.* **299**, 539–543 (1994).
44. Kaasik, A., Safiulina, D., Zharkovsky, A. & Veksler, V. Regulation of mitochondrial matrix volume. *Am J Physiol Cell Physiol* **292**, 157–163 (2007).

45. Moore, R. D. & Morrill, A. A POSSIBLE MECHANISM FOR CONCENTRATING SODIUM AND POTASSIUM IN THE CELL NUCLEUS. *Biophys. J.* **16**, 527–533 (1976).
46. Stellwagen, E. & Stellwagen, N. C. Probing the electrostatic shielding of DNA with capillary electrophoresis. *Biophys. J.* **84**, 1855–1866 (2003).
47. Attwood, S. J., Choi, Y. & Leonenko, Z. Preparation of DOPC and DPPC supported planar lipid bilayers for atomic force microscopy and atomic force spectroscopy. *Int. J. Mol. Sci.* **14**, 3514–3539 (2013).
