# Supplementary material for: Cations Regulate Membrane Attachment and Functionality of DNA Nanostructures
Source: J Am Chem Soc. 2021 May 7;143(19):7358–67. doi: 10.1021/jacs.1c00166 (PMC8154537; doi:10.1021/jacs.1c00166)
Supplement: Supplementary file 2 — ja1c00166_si_002.pdf [file ja1c00166_si_002.pdf]

Supplementary Movie 1: A video illustrating a 1.1 μs MD simulation of a dsDNA molecule interacting with a gel-phase (DPPE) lipid bilayer membrane. Periodic images of the system along both *y*-axis (left to wright) and *z*-axis (up and down) are shown to illustrate the binding of DNA across the periodic boundary of the simulation system. The backbone of the DNA is shown in green. The nucleotide bases of the 21-base pair DNA fragment in one simulation unit cell are shown in light blue whereas those from the periodic images are shown in white. Non-hydrogen atoms of the lipid membrane are shown as blue (N), tan (P), red (O), and cyan (C) spheres. Water and ions are not shown for clearly.

Supplementary Movie 2: A video illustrating a 1.2 μs MD simulation of a dsDNA molecule interacting with a fluid-phase (DPhPE) lipid bilayer membrane. Periodic images of the system along both *y*-axis (left to wright) and *z*-axis (up and down) are shown to illustrate the binding of DNA across the periodic boundary of the simulation system. The backbone of the DNA is shown in green. The nucleotide bases of the 21-base pair DNA fragment in one simulation unit cell are shown in light blue whereas those from the periodic images are shown in white. Non-hydrogen atoms of the lipid membrane are shown as blue (N), tan (P), red (O), and cyan (C) spheres. Water and ions are not shown for clarity.

Supplementary Movie 3: A video illustrating the strong binding of dsDNA molecule to a gel-phase (DPPE) lipid bilayer membrane. The video covers the last 90 ns of the equilibrium MD run (same as Movie 1) but explicitly shows the magnesium ions and their first solvation shell, $Mg[H_2O]_6^{2+}$, depicted as red and white spheres.

Supplementary Movie 4 : A video illustrating Mg^{2+}-mediated binding of dsDNA to a fluid-phase (DPhPE) lipid bilayer membrane. The video covers the last 100 ns of the

equilibrium MD run (same as Movie 2) but explicitly shows the magnesium ions and their first solvation shell, $Mg[H_2O]_6^{2+}$, depicted as red and white spheres.
